# Impacts of Supplementation with Silymarin on Cardiovascular Risk Factors: A Systematic Review and Dose–Response Meta-Analysis

**DOI:** 10.3390/antiox13040390

**Published:** 2024-03-24

**Authors:** Shooka Mohammadi, Omid Asbaghi, Reza Afrisham, Vida Farrokhi, Yasaman Jadidi, Fatemeh Mofidi, Damoon Ashtary-Larky

**Affiliations:** 1 Department of Social and Preventive Medicine, Faculty of Medicine, University of Malaya, Kuala Lumpur 50603, Malaysia; 2 Cancer Research Center, Shahid Beheshti University of Medical Sciences, Tehran 1416753955, Iran; omid.asbaghi@gmail.com; 3 Department of Clinical Laboratory Sciences, Faculty of Allied Medicine, Tehran University of Medical Sciences, Tehran 14176-13151, Iran; rafrisham@sina.tums.ac.ir (R.A.); y-jadidi@farabi.tums.ac.ir (Y.J.); 4 Department of Hematology, Faculty of Allied Medicine, Tehran University of Medical Sciences, Tehran 1417613151, Iran; v-farrokhi@razi.tums.ac.ir; 5 Department of Clinical Nutrition and Dietetics, National Nutrition and Food Technology Research Institute, Faculty of Nutrition and Food Technology, Shahid Beheshti University of Medical Sciences, Tehran 1416753955, Iran; dr.fatemehmofidi@gmail.com; 6 Nutrition and Metabolic Diseases Research Center, Ahvaz Jundishapur University of Medical Sciences, Ahvaz 6135715794, Iran

**Keywords:** silymarin, cardiometabolic syndrome (CMS), blood pressure (BP), glycemic parameters, lipid profile

## Abstract

It has been suggested that silymarin (SIL) supplementation has positive effects on cardiovascular health and reduces the risk of cardiometabolic syndrome (CMS). This systematic review and dose–response meta-analysis assessed the impacts of SIL administration on cardiovascular risk factors. A systematic search of multiple databases was performed to identify eligible controlled trials published up to January 2023. The analysis used a random-effects model and included 33 trials with 1943 participants. It was revealed that SIL supplementation led to a notable reduction in serum levels of fasting blood glucose (FBG) (weighted mean difference (WMD): −21.68 mg/dL, 95% CI: −31.37, −11.99; *p* < 0.001), diastolic blood pressure (DBP) (WMD: −1.25 mmHg; 95% CI: −2.25, −0.26; *p* = 0.013), total cholesterol (TC) (WMD: −13.97 mg/dL, 95% CI: −23.09, −4.85; *p* = 0.003), triglycerides (TG) (WMD: −26.22 mg/dL, 95% CI: −40.32, −12.12; *p* < 0.001), fasting insulin (WMD: −3.76 mU/mL, 95% CI: −4.80, −2.72; *p* < 0.001), low-density lipoprotein (LDL) (WMD: −17.13 mg/dL, 95% CI: −25.63, −8.63; *p* < 0.001), and hemoglobin A1C (HbA1c) (WMD: −0.85%, 95% CI: −1.27, −0.43; *p* < 0.001) in the SIL-treated groups compared to their untreated counterparts. In addition, there were no substantial differences in body mass index (BMI), systolic blood pressure (SBP), C-reactive protein (CRP), body weight, and high-density lipoprotein (HDL) between the two groups. These outcomes suggest that SIL consumption reduces certain CMS risk factors and has favorable impacts on lipid and glycemic profiles with potential hypotensive effects. These findings should be supported by additional trials with larger sample sizes and longer durations.

## 1. Introduction

Cardiometabolic syndrome (CMS) is a group of risk factors or metabolic disorders that include a combination of systemic arterial hypertension, diabetes mellitus, hyperlipidemia, and central obesity [1]. These metabolic conditions are linked to the onset of atherosclerotic cardiovascular disease (ASCVD) [2,3]. Antidiabetic, antihypertensive, lipid-lowering, and anti-obesity medications are the main focus of routine CMS management [4]. There is a growing interest in complementary and alternative medicine (CAM), such as herbal medicine, to treat metabolic dysfunction due to the persistent degenerative nature and prolonged therapy of CMS, its potential adverse effects, and its considerable economic burden [5]. In addition, herbal medicine has received growing interest in developing better therapeutic strategies for secondary or primary prevention of cardiovascular diseases (CVDs) [6,7]. Evidence supports the beneficial effects of silymarin (SIL) as a medicinal herb on cardiometabolic risk factors [8,9,10] with anti-atherosclerotic actions [11].

Milk thistle (*Silybum marianum* (L.) Gaertn) is a spiny herb that has been used as a herbal remedy and functional food ingredient for centuries [12,13]. Dried milk thistle seeds were used to extract SIL [14]. Silymarin is a polyphenolic flavonoid with 20–35% fatty acids, 70–80% SIL flavonolignans, and several other polyphenolic ingredients [15]. Silibinin (silybin) is the most prevalent and biologically active flavonoid isomer of SIL [13]. It is most commonly used for medicinal purposes to treat or protect the liver from toxic substances [12,16,17,18]. Several studies have revealed the cardioprotective and anti-obesity properties of SIL, as well as the beneficial impacts of SIL on CMS and lipid profiles [11,19,20,21]. Most of its potential therapeutic effects are related to its antioxidative characteristics [22]. Dietary antioxidant supplements may exert modulatory effects on oxidative stress (OS) [23]. Numerous clinical trials and reviews have been conducted to confirm the antioxidant effects of SIL and the effectiveness of its administration for the treatment of several medical disorders [22,24,25,26,27]. 

Silymarin may have atheroprotective effects and reduce LDL oxidation, which is a stage in the progression of atherosclerosis [11,28]. It may enhance vascular function [29,30] and have cardioprotective effects [31,32]. It could increase high-density lipoprotein (HDL) levels, but its effect on total cholesterol levels is debatable [33,34]. Furthermore, it has the potential to reduce levels of fasting blood glucose (FBG), triglyceride (TG), blood pressure (BP), and body weight [33,34,35,36]. It may decrease overall OS levels with a specific focus on inhibiting the release of oxidants by the liver [35,37]. The hepatoprotective effects of SL may positively influence various metabolic parameters [11,38]. Specifically, SIL can inhibit the generation of inflammatory agents from the liver, which play a significant role in the development of CVDs [11,39]. Several systematic reviews and meta-analyses have documented the positive impact of SIL on metabolic status [26], type 2 diabetes mellitus (T2DM) [24,40], and the features of CMS in adults [9,41]. However, the impact of SIL supplements on cardiovascular health in various consumer or patient groups has not been thoroughly evaluated, and the results are inconclusive, contradictory, and inconsistent. Therefore, this systematic review and dose–response meta-analysis of trials assessed the impacts of supplementation with SIL on cardiovascular risk factors in adults. 

## 2. Materials and Methods

The systematic review and meta-analysis protocol was registered in the international prospective register of systematic reviews (PROSPERO) with the registration number CRD42023488215. This procedure followed the guidelines outlined in the preferred reporting items for systematic reviews and meta-analyses (PRISMA) checklist [42].

### 2.1. Search Strategy

One investigator employed a systematic search using a systematic approach to find potentially relevant studies published until January 2023 in several databases (Scopus, Cochrane Library, PubMed, and Web of Science). Two researchers (V.F. and Y.J.) independently assessed the articles and selected eligible randomized controlled trials (RCTs) with crossover or parallel designs. The search strategy was formulated based on four essential elements in RCTs, encompassing population (adult), intervention/exposure (SIL supplement), comparator/control (placebo or intervention), and outcomes (body mass index (BMI), diastolic blood pressure (DBP), body weight, systolic blood pressure (SBP), serum levels of low-density lipoprotein (LDL), fasting blood glucose (FBG), C-reactive protein (CRP), triglycerides (TG), hemoglobin A1_C_ (HbA1c), total cholesterol (TC), fasting insulin, and high-density lipoprotein (HDL)). The search terms were as follows: (“silymarin” OR “silibinin” OR “silybin”) AND (“Intervention” OR “Intervention Study” OR “Intervention Studies” OR “controlled trial” OR “randomized” OR “randomised” OR “random” OR “randomly” OR “placebo” OR “clinical trial” OR “randomized controlled trial” OR “randomized clinical trial” OR “RCT” OR “blinded” OR “double blind” OR “double blinded” OR “trial” OR “trials” OR “Pragmatic Clinical Trial” OR “Cross-Over Studies” OR “Cross-Over” OR “Cross-Over Study” OR “parallel” OR “parallel study” OR “parallel trial”) AND (“lipid” OR “glycemic” OR “anthropometric” OR “body weight” OR “SBP” OR “DBP” OR “HbA1c” OR “LDL” OR “HDL” OR “TC” OR “FBG” OR “TG” OR “diastolic blood pressure” OR “systolic blood pressure” OR “body mass index” OR “fasting blood glucose” OR “insulin” OR “hemoglobin A1_C_” OR “low-density lipoprotein” OR “total cholesterol” OR “C-reactive protein” OR “CRP” OR “high-density lipoprotein” OR “low-density lipoprotein” OR “triglycerides”).

### 2.2. Eligibility Criteria 

The researchers employed EndNote software (version 20) to facilitate the export of articles. Subsequently, two researchers (V.F. and Y.J.) independently checked the abstracts with the titles of the articles and extracted the pertinent data from the eligible full-text articles. Any discrepancies were addressed through collaborative discussions with a third researcher (D.A.L.). The current systematic review and meta-analysis encompassed all trials that investigated the efficacy of supplementation with SIL on body weight, BMI, SBP, DBP, serum levels of FBG, HbA1c, TG, fasting insulin, LDL, CRP, TC, and HDL in the SIL group compared to the placebo group.

Eligible RCTs enrolled adult participants; they had control or placebo groups and presented sufficient data on predetermined outcomes in the SIL-treated and untreated groups at the study endpoint and baseline. Furthermore, SIL was not administered as a multi-component supplement. Studies that did not have the following criteria were omitted from the analysis: uncontrolled trials; observational studies utilizing case–control, cross-sectional, or cohort designs; non-peer-reviewed articles or abstracts; studies that involved pregnant women or individuals under 18 years old; articles that did not report the impact of SIL administration on the specified outcomes compared to the control group; and studies with insufficient data on baseline or follow-up measurements of body weight, BMI, SBP, DBP, serum levels of HDL, fasting insulin, FBG, HbA1c, LDL, TC, TG, and CRP.

### 2.3. Data Extraction

Two researchers independently extracted the data (S.M. and Y.J.). Information regarding the study’s characteristics (e.g., study design, duration and location of the trial, sample size, first author’s name, publication year, and dosage of SIL supplement), participants’ demographic details (e.g., gender, average age, BMI, and comorbidities), initial and final measurements of specific outcomes, and mean differences in outcome changes between the study’s initial and final measurements. The main outcomes were body weight, BMI, SBP, DBP, and serum levels of TG, fasting insulin, HbA1c, LDL, CRP, FBG, TC, and HDL. In addition, two researchers resolved disagreements and discrepancies through discussions.

### 2.4. Quality Assessment

Two separate researchers (S.M. and D.A.L.) evaluated the quality of the trials using the Cochrane risk of bias (RoB 2) tool [43]. It determined potential sources of bias, including reporting bias, performance bias, attrition bias, detection bias, and allocation bias. The RoB for each domain was categorized as low, unclear, or high [43].

### 2.5. Statistical Analysis 

This meta-analysis used Stata software (version 17). The impact of SIL was assessed through the calculation of weighted mean differences (WMDs) along with 95% confidence intervals (CIs) to measure the absolute changes in outcomes between the SIL and placebo groups from the study baseline to endpoints. The outcomes were quantified using the mean ± standard deviation (SD) measurement, and the effect size was identified by calculating the mean difference. The subsequent formula was employed to compute the SD change from the SD at the baseline of the trial to the SD at the end of the trial: square root ([SD^2^_baseline_ + SD^2^_final_] − [2 × R × SD_baseline_ × SD_final_]) [44]. Furthermore, a random-effects model [45] was applied to determine the pooled WMDs. The between-study heterogeneity was assessed using the I^2^ statistic [46]; I^2^ values of 50%, 25%, and 75% were categorized as representing moderate, low, and high levels of heterogeneity, respectively [47].

Subgroup analyses were carried out to explore the potential factors contributing to the heterogeneity across trials. The analysis considered factors such as baseline values of serum levels of HDL, TC, FBG, TG, LDL, and CRP, as well as the dosage of SIL (≥400 mg/day vs. <400 mg/day), baseline BMI (obese (≥30 kg/m^2^) vs. overweight (25–29.9 kg/m^2^) vs. normal (18.5–24.9 kg/m^2^)), trial duration (<12 weeks vs. ≥12 weeks), and health status (healthy vs. unhealthy). A leave-one-out sensitivity analysis was applied to assess the effects of individual studies on the analysis. Statistical significance was considered at a level of *p* < 0.05. In addition, funnel plots and Begg’s [48] and Egger’s tests [49] were utilized to identify possible publication bias. Additionally, the fractional polynomial model was employed to investigate the potential non-linear effects of the duration of the trial (weeks) and the dosage of SIL (mg/day). Meta-regression analysis was implemented to assess the dose–response relationship and potential linear associations between duration trials, SIL dosage, and effect sizes [50].

### 2.6. GARDE Assessment

The certainty of the evidence was evaluated using the GRADE framework (Grading of Recommendations Assessment, Development, and Evaluation) categorized into four levels (moderate, high, very low, and low) [51].

## 3. Results

### 3.1. Study Selection

**Figure 1:**
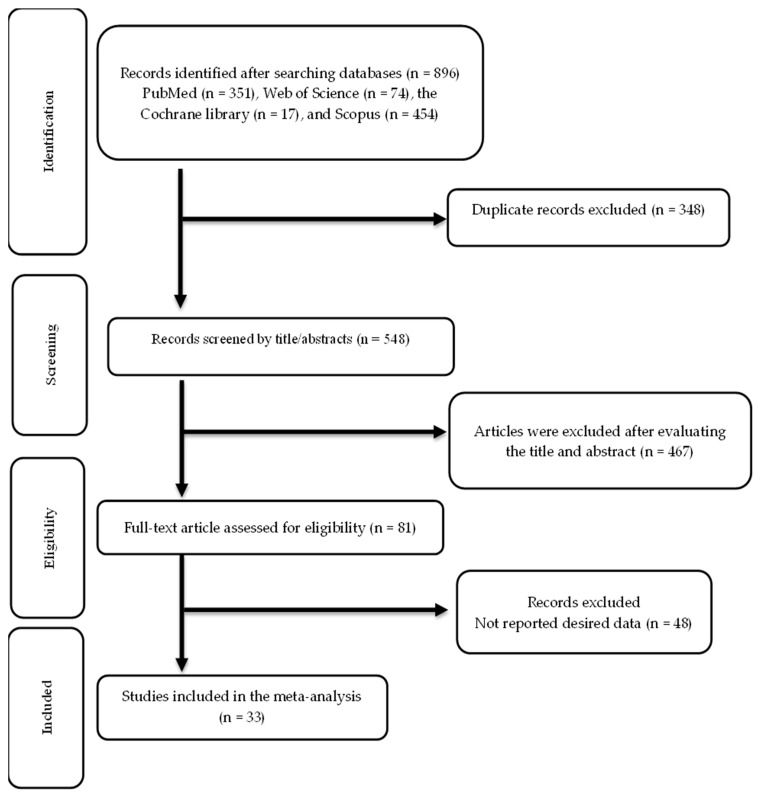
Flow chart of study selection.

The screening and selection processes for the included trials are illustrated in Figure 1. The first search across multiple databases yielded 896 publications. After eliminating 348 duplicate records, 548 records were reviewed, and 467 were not relevant. The remaining 81 articles were assessed for eligibility, and 33 articles were ultimately included in this meta-analysis [52,53,54,55,56,57,58,59,60,61,62,63,64,65,66,67,68,69,70,71,72,73,74,75,76,77,78,79,80,81,82,83,84].

### 3.2. Study Characteristics

This meta-analysis included 33 RCTs with 1943 participants (978 cases and 982 controls). Characteristics of the included trials are provided in Table 1. The articles were published from 1993 to 2022. Thirty-two studies used a randomized parallel design [52,53,54,55,56,57,58,59,60,61,62,63,64,65,66,67,68,69,70,71,72,73,74,75,76,77,79,80,81,82,83,84], while one study was a cross-over trial [78]. The trial duration and sample size varied from 2 to 48 weeks and 16 to 100 participants, respectively. The mean age of the participants varied from 20 to 63 years old. The trials were performed in Iran [55,56,59,60,61,62,64,65,67,68,69,70,72,73,75,76,77,79,81,82], Italy [52,53,84], the Czech Republic [54,58], Iraq [57,63,66], Malaysia [74], Egypt [71], Pakistan [80,83], and Australia [78]. They were conducted among healthy individuals [54,72,73,78], patients with cirrhosis [52,53], diabetic nephropathy [64], diabetes mellitus [55,56,57,59,63,65,67,70,71,75,77,79,80,83,84], metabolic syndrome [58], hyperlipidemia [66], β-thalassemia major (β-TM) [61], non-alcoholic fatty liver disease (NAFLD) [60,69,76,82], non-alcoholic steatohepatitis [62,68,74], and coronavirus disease 2019 (COVID-19) [81]. One study was implemented among women [79] and five among men [54,72,73,77,78], whereas the majority of the trials comprised both sexes. The daily intake of SIL supplements varied between 140 and 2100 mg. Supplementary Table S1 presents the RoB assessments of trials.

### 3.3. Meta-Analysis

#### 3.3.1. Impact of Silymarin Supplementation on Anthropometric Parameters

##### BMI

**Figure 2:**
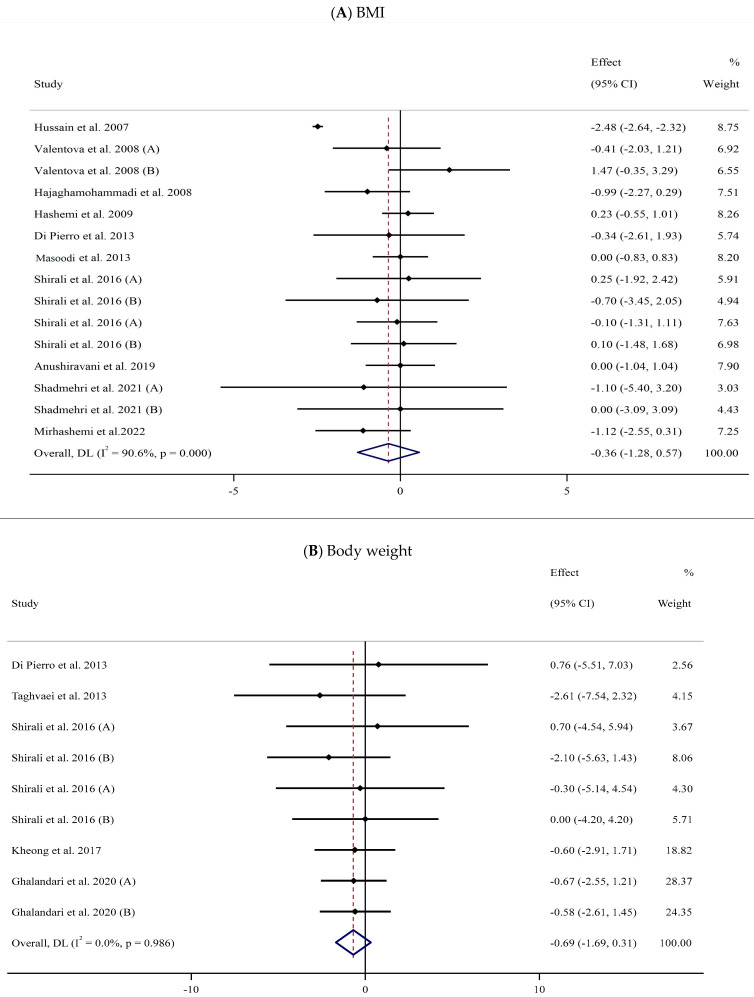

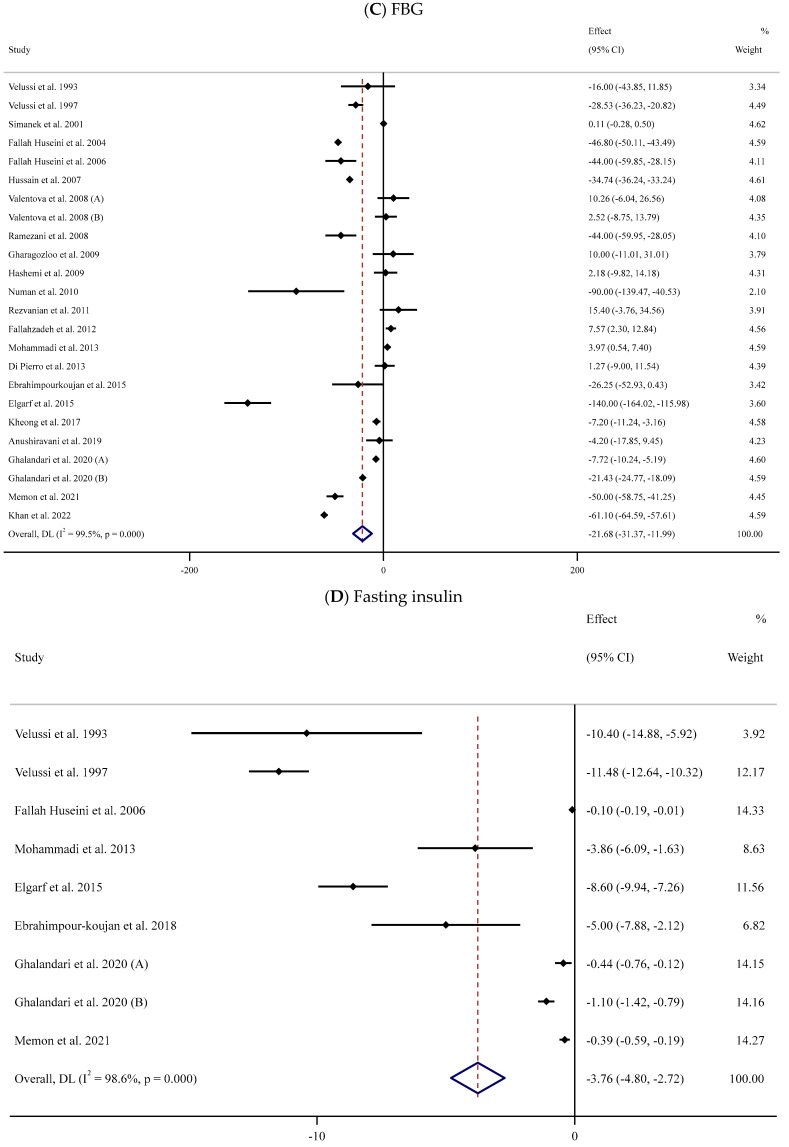

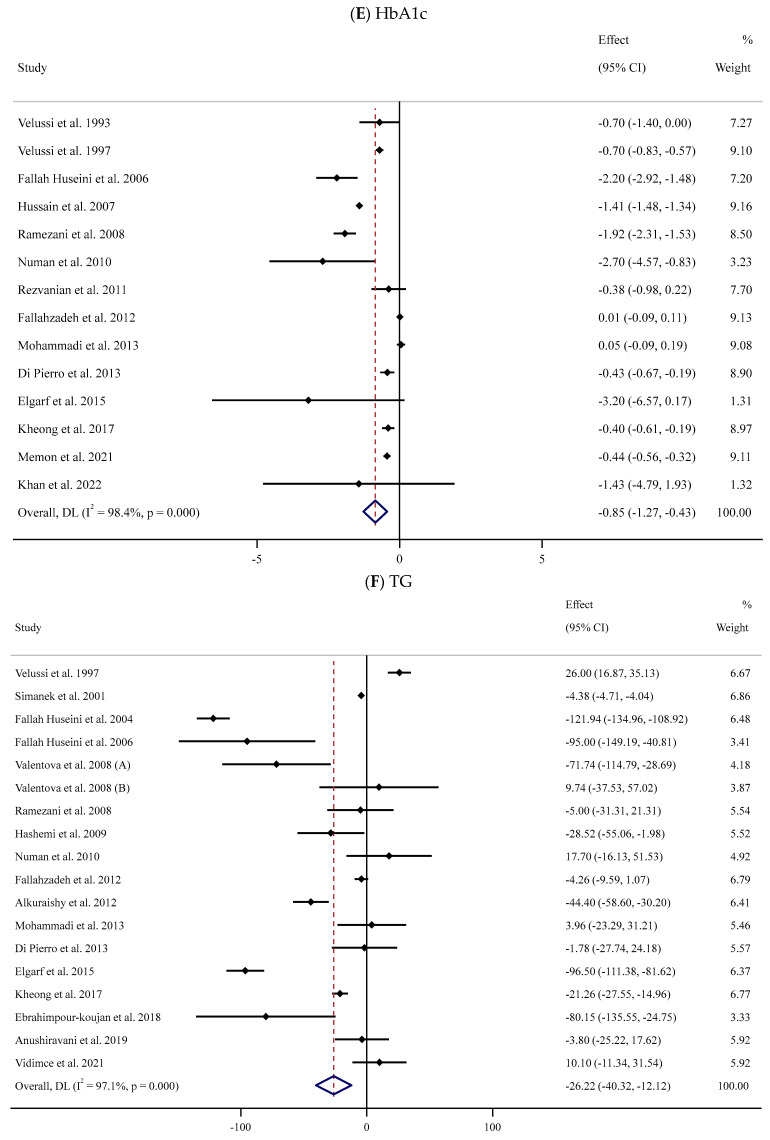

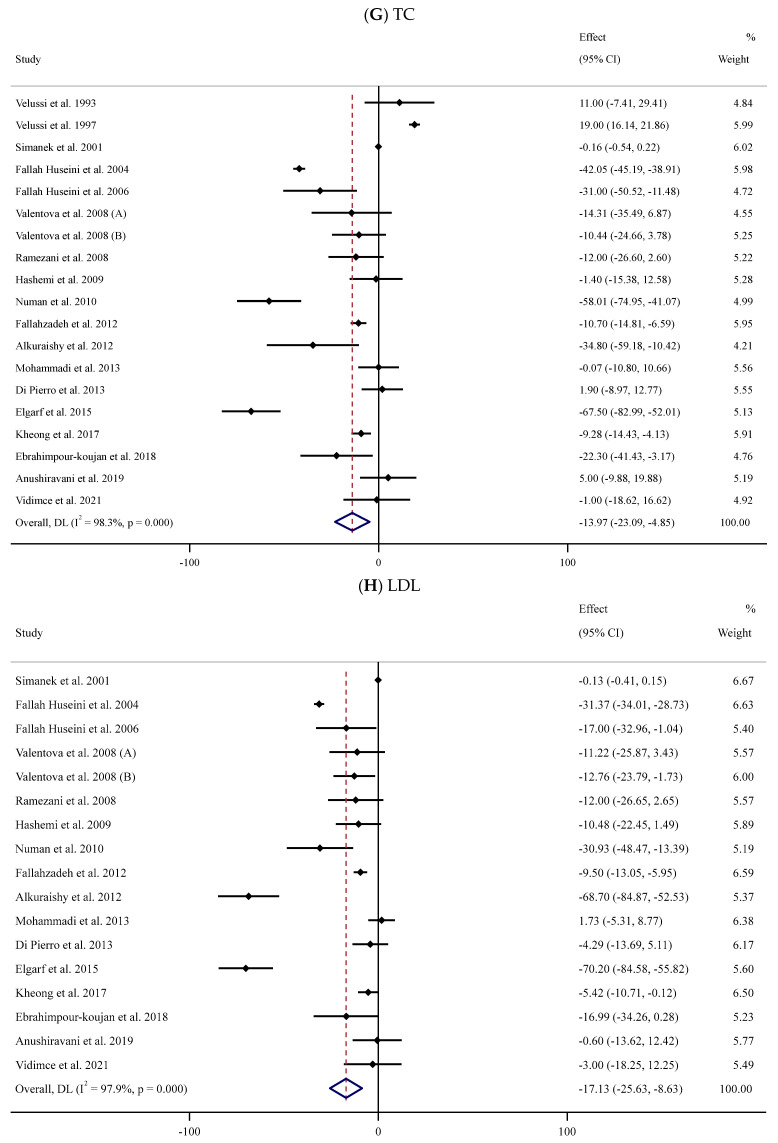

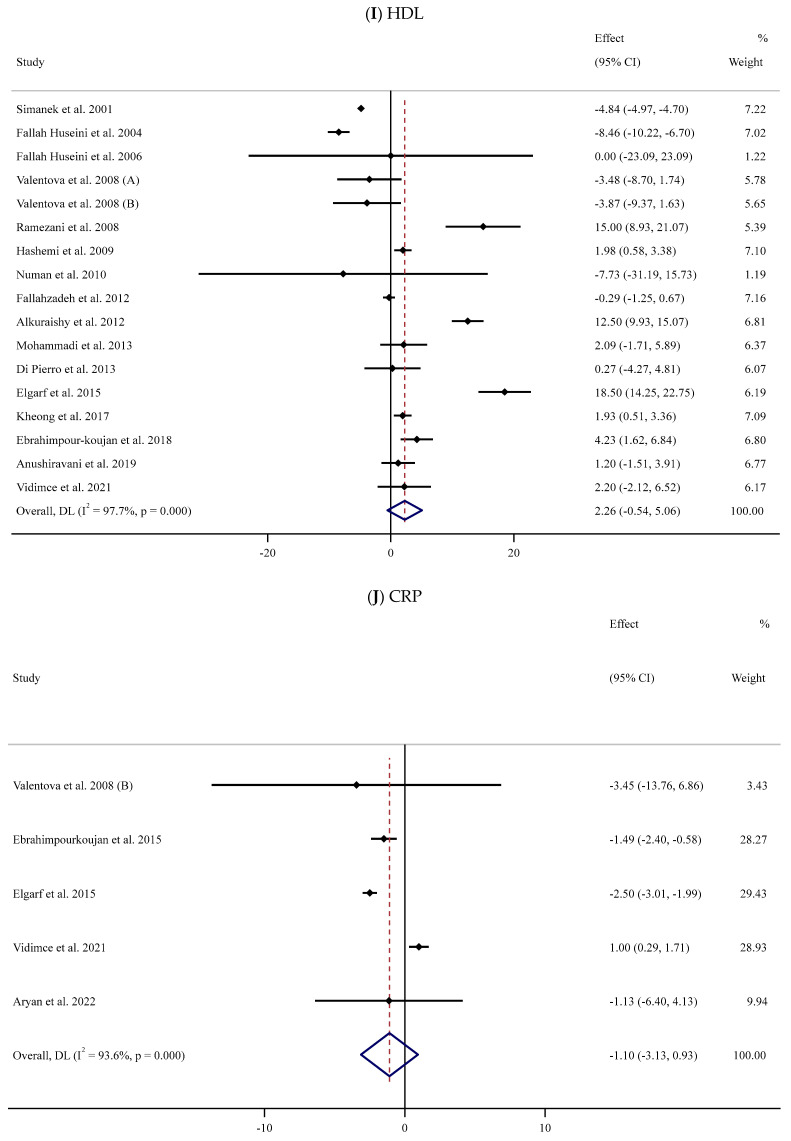

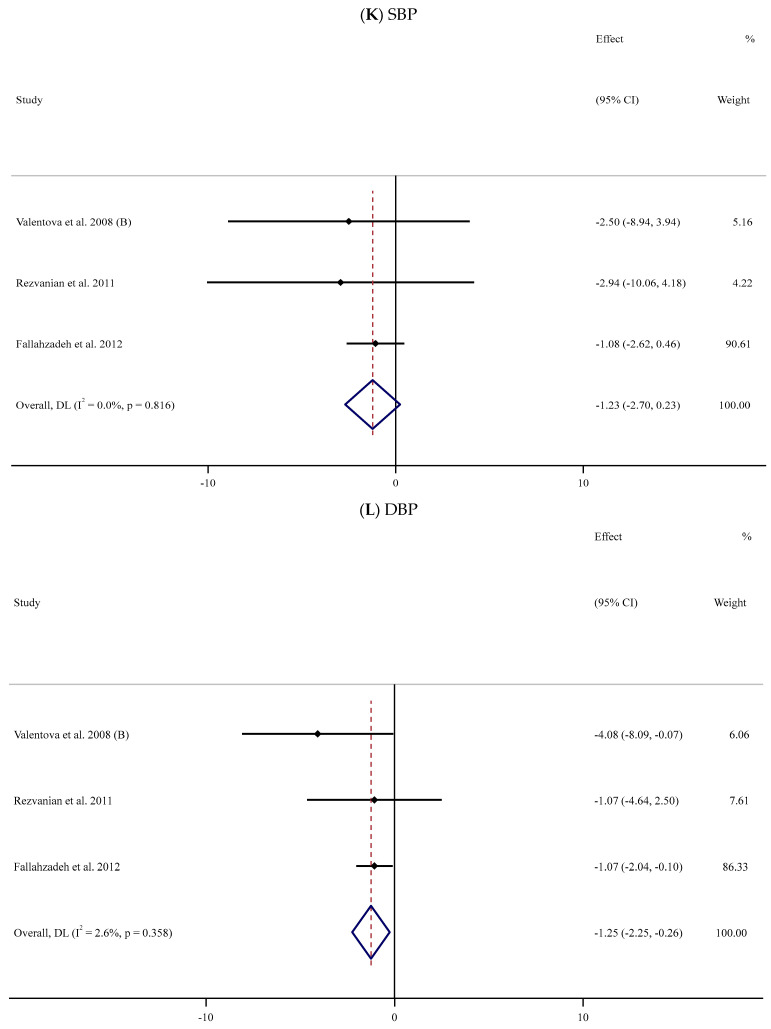
Forest plots for the effect of silymarin supplementation on (**A**) BMI (kg/m^2^), (**B**) body weight (kg), (**C**) FBG (mg/dL), (**D**) fasting insulin (mU/mL), (**E**) HbA1c (%), (**F**) TG (mg/dL), (**G**) TC (mg/dL), (**H**) LDL (mg/dL), (**I**) HDL (mg/dL), (**J**) CRP (mg/dL), (**K**) SBP (mmHg), and (**L**) DBP (mmHg) [52,53,54,55,56,57,58,59,60,61,62,63,64,65,66,67,68,69,70,71,72,73,74,75,76,77,78,79,80,81,82,83,84]. Horizontal lines represent 95% confidence intervals (CIs). Diamonds represent pooled estimates from random-effects analysis. The effect column comprises weighted mean differences (WMDs) and 95% CIs.

Eleven trials [57,58,60,62,69,72,73,76,79,82,84] with 15 arms and 671 participants (SIL-treated, *n* = 327; untreated, *n* = 344) were analyzed to evaluate the impact of SIL supplementation on BMI (Figure 2A). Analysis of the pooled data displayed no considerable differences in BMI between the two groups (WMD: −0.36 kg/m^2^, 95% CI: −1.28, 0.57; *p* = 0.447) with substantial heterogeneity (I^2^ = 90.6%, *p* < 0.001). The subgroup analysis explored a significant fall in BMI among participants with obesity (Table 2).

##### Body Weight

The impact of SIL intake on body weight was investigated in six studies [68,72,73,74,77,84] with 325 participants (Table 2). A pooled analysis of nine effect sizes indicated no substantial differences in body weight between the SIL-treated and untreated groups (WMD: −0.69 kg, 95% CI: −1.69, 0.31; *p* = 0.178, I^2^ = 0.0%) (Figure 2B).

#### 3.3.2. Impact of Silymarin Supplementation on Glycemic Parameters

##### FBG

The effect of supplementation with SIL on the concentrations of serum FBG was assessed in 22 trials [52,53,54,55,56,57,58,59,61,62,63,64,65,67,70,71,74,76,77,80,83,84] that involved 1445 participants (719 cases versus 726 controls). The pooled results indicated that SIL effectively decreased serum FBG values in the SIL-treated groups (*n* = 719) compared to their untreated counterparts (*n* = 726) (WMD: −21.68 mg/dL, 95% CI: −31.37, −11.99; *p* < 0.001) with remarkable between-trial heterogeneity (I^2^ = 99.5%, *p* < 0.001) (Figure 2C). Subgroup analyses revealed a considerable decrease in serum levels of FBG in the SIL group in comparison to the untreated group in studies involving unhealthy individuals with obesity and baseline serum FBG levels > 126 mg/dL. This reduction was observed in long- and short-term supplementation (<12 and ≥12 weeks) with high or low doses of SIL (≥400 or <400 mg/day) (Table 2).

##### Fasting Insulin 

The association between serum fasting insulin levels and SIL intake was assessed in eight studies [52,53,56,67,71,75,77,80] involving 571 participants (Figure 2D). The meta-analysis revealed that serum fasting insulin concentrations decreased following SIL consumption compared to the untreated group (WMD: −3.76 mU/mL, 95% CI: −4.80, −2.72; *p* < 0.001); nevertheless, there was substantial heterogeneity between trials (I^2^ = 98.6%, *p* < 0.001) (Table 2). Subgroup analyses indicated a notable reduction in serum levels of insulin in the SIL-treated group compared to controls in studies among participants with overweight or obesity, in short-term or long-term supplementation with low or high doses of SIL.

##### HbA1c

Fourteen studies [52,53,56,57,59,63,64,65,67,71,74,80,83,84] assessed the impact of supplementation with SIL on the serum HbA1c levels in a total of 957 participants (SIL-treated vs. placebo). Meta-analysis of these trials indicated a substantial decline in serum HbA1c levels following SIL intake compared to the untreated group (WMD: −0.85%, 95% CI: −1.27, −0.43; *p* < 0.001) with a high degree of between-trial heterogeneity (I^2^ = 98.4%, *p* < 0.001) (Figure 2E). Subgroup analyses suggested a considerable fall in serum HbA1c concentrations in the SIL group in comparison with the untreated group in trials with short- or long-term duration and high daily doses of SIL among participants with obesity (Table 2).

#### 3.3.3. Impact of Silymarin Supplementation on Lipid Profile

##### TG

The meta-analysis of 17 RCTs [53,54,55,56,58,59,62,63,65,66,67,71,74,75,76,78,84] with 953 participants (480 cases and 490 controls) indicated a substantial reduction in serum TG levels in the SIL group in comparison with the controls (WMD: −26.22 mg/dL, 95% CI: −40.32, −12.12; *p* < 0.001). Furthermore, there was considerable heterogeneity among trials (I^2^ = 97.1%, *p* < 0.001) (Figure 2F). Similar findings were detected in subgroup analyses among unhealthy participants with normal BMI or obesity and baseline serum TG levels >150 mg/dL after long-term supplementation with high doses of SIL (Table 2).

##### TC

Eighteen trials [52,53,54,55,56,58,59,62,63,65,66,67,71,74,75,76,78,84] with 1013 participants (SIL group vs. controls) explored the impact of SIL administration on serum TC levels (Figure 2G). Pooled analysis showed that SIL consumption caused a significant decline in serum TC levels (WMD: −13.97 mg/dL, 95% CI: −23.09, −4.85; *p* = 0.003) with substantial between-trial heterogeneity (I^2^ = 98.3%, *p* < 0.001). Subgroup analyses indicated a notable reduction in serum TC concentration among unhealthy participants with obesity and baseline serum TC levels >200 or <200 mg/dL after short-term supplementation with high doses of SIL (Table 2).

##### LDL

Sixteen RCTs [54,55,56,58,59,62,63,65,66,67,71,74,75,76,78,84] with 893 participants (450 cases vs. 460 controls) were included in the meta-analysis to evaluate the impact of SIL consumption on serum LDL levels (SIL group vs. placebo group). Analysis of the pooled effect sizes revealed a significant decline in serum LDL concentration in the SIL-treated group (WMD: −17.13 mg/dL, 95% CI: −25.63, −8.63; *p* < 0.001) with substantial heterogeneity (I^2^ = 97.9%, *p* < 0.001) (Figure 2H). Similar results were revealed based on sub-analyses among overweight unhealthy participants with high or low baseline serum LDL levels during short- or long-term supplementation with high doses of SIL (Table 2).

##### HDL

Sixteen trials [54,55,56,58,59,62,63,65,66,67,71,74,75,76,78,84] were conducted to assess the impact of SIL on serum HDL levels among 893 participants. The meta-analysis revealed that there was no substantial impact of SIL on the HDL level in the SIL-treated group when compared to the control group (WMD: 2.26 mg/dL, 95% CI: −0.54, 5.06; *p* = 0.114). Considerable heterogeneity between studies was also noted (I^2^ = 97.7%, *p* < 0.001) (Figure 2I). Subgroup analyses revealed a substantial reduction in serum HDL levels among participants with normal BMI and high or low baseline serum HDL levels after supplementation with low doses of SIL (Table 2).

#### 3.3.4. Impact of Silymarin Supplementation on Serum CRP 

In the meta-analysis of five RCTs [58,70,71,78,81] with 185 participants, SIL consumption had no substantial impact on serum CRP levels (WMD: −1.10 mg/dL, 95% CI: −3.13, 0.93; *p* = 0.289) (Figure 2J). However, the outcomes showed considerable between-study heterogeneity (I^2^ = 93.6%, *p* < 0.001) (Table 2). Subgroup analyses revealed a substantial decline in serum CRP levels among healthy and unhealthy participants with obesity and baseline serum CRP levels >3 mg/L in long-term (≥12 weeks) supplementation with high doses of SIL (Table 2).

#### 3.3.5. Impact of Silymarin Supplementation on Blood Pressure

##### SBP and DPB 

The impact of supplementation with SIL on BP (SBP and DBP) was analyzed in three trials [58,64,65] involving 150 participants (SIL group, *n* = 78; control group, *n* = 72) (Table 2). The pooled data revealed no statistically notable differences in SBP values between the two groups (WMD: −1.23 mmHg, 95% CI: −2.70, 0.23; *p* = 0.099, I^2^ = 0%) (Figure 2K); however, a significant reduction in the magnitude of DBP was observed among the SIL-treated participants in comparison with the placebo group (WMD: −1.25 mmHg; 95% CI: −2.25, −0.26; *p* = 0.013, I^2^ = 2.6%) (Figure 2L).

### 3.4. Publication Bias 

Begg’s and Egger’s tests did not display any substantial publication bias for several evaluated outcomes (body weight, DBP, serum levels of TC, HbA1c, TG, and CRP). However, evidence of potential bias was detected in trials assessing the effects of SIL consumption on BMI (Begg’s test, *p* = 0.018; Egger’s test, *p* <0.001), SBP (Egger’s test, *p* = 0.046), serum levels of HDL (Egger’s test, *p* = 0.002), LDL (Egger’s test, *p* = 0.022), FBG (Egger’s test, *p* = 0.036), and insulin (Egger’s test, *p* = 0.013). Additionally, a visual examination of funnel plots revealed different degrees of asymmetry for all outcomes (Supplementary Figure S1).

### 3.5. Sensitivity Analysis

Sensitivity analyses revealed that the findings were not influenced by the removal of any specific trial related to body weight, BMI, SBP, and serum levels of insulin, FBG, HbA1c, TG, TC, LDL, and CRP. However, the outcomes related to DBP values changed after excluding two studies [64,65]. In addition, the outcomes related to serum HDL values changed after excluding one study [55].

### 3.6. GRADE Assessment

The GRADE framework was utilized to evaluate the quality of evidence for all outcomes (Supplementary Table S2). The certainty of evidence related to DBP values was deemed low due to a very serious risk of bias. For the other assessed outcomes, GRADE was rated as very low, primarily attributed to serious risks of imprecision, publication bias, very serious inconsistency, and bias.

### 3.7. Linear and Non-Linear Dose–Response Associations

No linear (Supplementary Figures S4 and S5) or non-linear (Supplementary Figures S2 and S3) correlation was depicted between the duration of the trial or the dosage of SIL supplement and the alterations in all assessed outcomes.

## 4. Discussion

This systematic review and dose–response meta-analysis examined the impact of supplementation with SIL on CVD risk factors. The findings suggest that SIL intake may improve certain CMD risk factors by decreasing levels of FBG, fasting insulin, HbA1c, TC, TG, LDL, and DBP. However, it did not significantly affect anthropometric measurements, SBP, or serum CRP levels. Supplementation with SIL demonstrated enhancements in glycemic control, lipid profile, and potential hypotensive effects. 

The subgroup analysis revealed a substantial decrease in BMI among individuals with obesity following SIL supplementation compared to their untreated counterparts. It also indicated a notable reduction in FBG levels in trials involving unhealthy participants with obesity and baseline FBG levels >126 mg/dL during both long- and short-term supplementation (<12 and ≥12 weeks) with low or high doses of SIL (≥400 or <400 mg/d). Furthermore, a considerable decline in serum insulin levels was observed among participants with overweight or obesity following short- and long-term supplementation with low or high doses of SIL. It also suggested a significant fall in HbA1c concentration in trials with short- or long-term duration and high doses of SIL among participants with obesity. Additionally, a substantial reduction in TG levels was noted in unhealthy participants with a normal BMI or obesity and baseline serum TG levels below 150 mg/dL after long-term supplementation with high doses of SIL. It indicated a significant decrease in serum TC concentration among unhealthy obese participants during short-term supplementation with high doses of SIL. A notable fall in serum LDL levels was detected in unhealthy overweight participants after receiving high doses of SIL over a short or long period. Furthermore, a considerable reduction in serum HDL levels was depicted in participants with a normal BMI after supplementation with low doses of SIL. In addition, there was a considerable decrease in serum levels of CRP among both healthy and unhealthy participants with obesity and baseline CRP levels >3 mg/L over long-term supplementation with high doses of SIL.

The outcomes of this study regarding serum lipids were in agreement with two previous meta-analyses [41,85]. Similarly, the results related to lipid profiles and glycemic parameters were consistent with those reported in another meta-analysis [26]. However, there were discrepancies when compared with a meta-analysis of seven trials among patients with T2DM, which indicated that SIL did not lead to a substantial decline in serum levels of TC and TG [40]. Furthermore, a meta-analysis comprising five trials demonstrated that SIL could lower FBS and HbA1c levels without affecting the lipid profile of T2DM patients [24]. Although one meta-analysis indicated a positive impact of SIL on HDL levels, the clinical significance of this effect was minimal [41], which aligns with the non-significant increase observed in the present study.

In recent years, there have been extensive investigations into the potential health benefits and cardiovascular-protective properties of SIL and its supplements. Previous studies in animals have indicated that SIL intake may reduce the risk of CVDs [86,87]. Silymarin has been shown to contribute to the restoration of pancreatic function [88]. Animal studies have demonstrated that SIL and its components have the potential to improve glucose regulation in diabetic rats [89,90]. It could promote the restoration of pancreatic function by upregulating insulin and glucagon proteins, leading to the normalization of blood glucose levels and the restoration of insulin serum levels [88]. It has been reported that supplementation with SIL may improve the restoration of Langerhans islet β cells [91], stimulation of beta precursor cells to develop into insulin-producing cells, and reduction of advanced glycation end product (AGE) formation [92]. Silymarin exhibits low water solubility and limited absorption following oral administration [93], potentially leading to insignificant responses in certain studies. Therefore, enhancing the bioavailability of SIL by consuming it with or after a meal may increase its effectiveness [85]. Additionally, evidence suggests that SIL may promote the proliferation of insulin-producing cells [94]. Silymarin was considered safe for human consumption at therapeutic levels and was well tolerated, even at a high dosage of 700 mg administered three times daily over 24 weeks [95].

The positive impacts of SIL on metabolic status are related to its antioxidant effects [28]. In addition, SIL may have beneficial impacts on blood lipids through various mechanisms [9,78,96,97,98,99,100]. Hypertension is a significant factor contributing to CVDs [101]. Existing evidence suggests that the mechanisms through which SIL influences the prevention and management of high BP can be categorized into various groups [102]. Silymarin plays a role in regulating vascular tone and suppressing platelet aggregation, thereby contributing to the regulation of BP in individuals with hypertension [102]. 

It has been indicated that incorporating antioxidant-rich foods into the diet may enhance the treatment of patients with T2DM [103]. Given the antioxidant properties of SIL, it is hypothesized that it may be particularly effective in improving CVD risk factors; however, further evidence is required to fully support this assertion. The present systematic review and meta-analysis demonstrated a considerable cardioprotective benefit of supplementation with SIL.

It is important to emphasize that in all clinical trials, participants received standard treatment in conjunction with SIL supplementation [41]. Consequently, the specific effects of SIL remain unclear [41]. However, discontinuing standard treatment for ethical reasons was not feasible, and only the synergistic effects were investigated. Furthermore, the stringent regulations governing the production of nutraceuticals and herbal and dietary supplements are less comprehensive than those for pharmaceuticals. This could potentially lead to ineffectiveness in some cases due to the limited examination of effective dosage, bioavailability, and formulation [104]. Since most studies did not provide detailed information on SIL supplements, addressing this aspect was not feasible in the current systematic review and meta-analysis [41]. 

This first dose–response meta-analysis examined the effect of SIL consumption on CVD risk factors. The selection of trials for the systematic search was not limited by period or language. Moreover, a sufficient number of trials was evaluated in the analysis. Additionally, supplementary analyses including subgroup analyses, publication bias assessments, and sensitivity analyses were conducted. Systematic reviews and meta-analyses are widely regarded as the most reliable forms of clinical evidence [104,105]. However, evidence regarding the efficacy of herbal dietary supplements remains inconclusive [106]. Key concerns related to herbal nutraceuticals include the absence of standardized extracts, ambiguity regarding plant parts, and failure to disclose the type of extract [106]. The absence of rigorous regulations poses challenges for nutraceutical manufacturers in verifying the quality, safety, and effectiveness of their products [106]. Consequently, numerous available products may be ineffective [106].

This meta-analysis encountered various limitations, including significant methodological and clinical heterogeneity among trials. This heterogeneity was evident in the varying trial durations, doses of SIL supplements, participants’ underlying health conditions, and sample sizes. The majority of trials had poor quality with a high risk of bias. Moreover, over half of the trials in this meta-analysis were performed in a single country (Iran), emphasizing the necessity for additional research in various geographical locations to validate positive results across different ethnic populations. In addition, the included trials involved diverse non-intervention or control groups. 

## 5. Conclusions

In summary, the results of this meta-analysis indicated that SIL may mitigate certain cardiometabolic risk factors by reducing FBG, fasting insulin, HbA1c, TC, TG, LDL, and DBP levels. However, it did not significantly affect the anthropometric parameters, SBP values, or serum CRP levels. It demonstrated positive effects on lipid and glycemic profiles with potential hypotensive impacts but did not affect anthropometric or inflammatory parameters. SIL has the potential to be a beneficial adjunctive treatment for enhancing various aspects of chronic CMS. However, the existing data show significant variability and a lack of extensive clinical trials for specific parameters. These findings should be validated by additional well-designed RCTs with larger sample sizes and longer durations to ascertain the clinical efficacy and potential therapeutic application of SIL supplementation in cardiovascular health.

## Figures and Tables

**Table 1:** Characteristic of included studies in meta-analysis.

Author, Year	Country	Study Design	Participants	Sex	Sample Size		Trial Duration (Week)	Mean Age		Mean BMI		SIL Dose (mg/d)	CG
					IG	CG		IG	CG	IG	CG		
Velussi et al., 1993 [52]	Italy	Parallel, R, CO	Cirrhotic diabetic patients	♂/♀	30	30	24	63 ± 21.9	62 ± 16.4	25.1 ± 1.1	24.9 ± 6.02	600	Non-placebo
Velussi et al., 1997 [53]	Italy	Parallel, R, CO	Cirrhotic diabetic patients	♂/♀	30	30	48	63 ± 4	62 ± 3	25.1 ± 1.1	24.9 ± 0.7	600	Non-placebo
Simanek et al., 2001 [54]	Czech Republic	Parallel, R, DB, PC	Healthy young men	♂	17	12	9	20.6 ± 0.8	20.7 ± 0.6	22.6 ± 0.9	21.6 ± 0.7	858	Placebo
Fallah Huseini et al., 2004 [55]	Iran	Parallel, R, DB, PC	Patients with T2DM and hyperlipidemia	♂/♀	29	25	16	51.2 ± 7.2	53.8 ± 6.7	NR	NR	600	Placebo
Fallah Huseini et al., 2006 [56]	Iran	Parallel, R, DB, PC	Patients with T2DM	♂/♀	25	26	16	53.0 ± 6.6	54.1 ± 6.0	NR	NR	600	Placebo
Hussain et al., 2007 [57]	Iraq	Parallel, R, DB, PC	Patients with T2DM	♂/♀	18	20	17	49.2 ± 4.8		31.6 ± 0.4	30.9 ± 0.3	200	Placebo
Valentova et al., 2008 (A) [58]	Czech Republic	Parallel, R, DB, PC	Patients with MetS	♂/♀	14	34	12	5.4 ± 6.9	50.9 ± 12.8	31.1 ± 4.4	28.6 ± 4.7	800	Placebo
Valentova et al., 2008 (B) [58]	Czech Republic	Parallel, R, DB, PC	Patients with MetS	♂/♀	19	19	12	51.4 ± 8.0	46.6 ± 8.7	27.8 ± 5.2	27.6 ± 3.7	200	Meca
Ramezani et al., 2008 [59]	Iran	Parallel, R, DB, PC	Patients with T2DM	♂/♀	30	30	8	54.6 ± 2.1	52.1 ± 3.4	NR	NR	600	Placebo
Hajaghamohammadi et al., 2008 [60]	Iran	Parallel, R, DB, PC	Patients with NAFLD	♂/♀	25	25	8	40.3	39.9	30	29.1	140	Placebo
Gharagozloo et al., 2009 [61]	Iran	Parallel, R, DB, PC	Patients with β-thalassemia major	♂/♀	29	30	12	20.2 ± 6.2	17.9 ± 3.7	17.2 ± 1.9	17.9 ± 1.8	420	Placebo
Hashemi et al., 2009 [62]	Iran	Parallel, R, DB, PC	Patients with NASH	♂/♀	50	50	24	39.2 ± 11.1	39.0 ± 10.7	26.75	27.8	280	Placebo
Numan et al., 2010 [63]	Iraq	Parallel, R, DB, PC	Patients with T1D	♂/♀	30	30	8	44.3 ± 16.4		NR	NR	400	Placebo
Rezvanian et al., 2011 [64]	Iran	Parallel, R, DB, PC	Patients with T2DM nephropathy	♂/♀	29	23	12	55.9 ± 6.5	57.7 ± 7.7	NR	NR	420	Placebo
Fallahzadeh et al., 2012 [65]	Iran	Parallel, R, DB, PC	Patients with T2DM	♂/♀	30	30	12	55.9 ± 8.3	57.6 ±7.5	28.6 ± 6	29.2 ± 4.8	420	Placebo
Alkuraishy et al., 2012 [66]	Iraq	Parallel, R, PC	Patients with hyperlipidemia	♂/♀	10	10	2	36–64		NR	NR	600	Placebo
Mohammadi et al., 2013 [67]	Iran	Parallel, R, DB, PC	Patients with T2DM	♂/♀	30	30	12	50.1 ± 13.1	45.4 ± 11.9	28.4 ± 3.3	29.19 ± 3.6	280	Placebo
Di Pierro et al., 2013 [84]	Italy	Parallel, R, SB, PC	Patients with T2DM	♂/♀	30	27	16	67.8 ± 10.8	66.3 ± 9.8	29.9 ± 7.2	30.5 ± 6.8	210	Berberis aristata
Taghvaei et al., 2013 [68]	Iran	Parallel, R, DB, PC	Patients with NASH	♂/♀	21	20	24	42.9 ± 11.42	40.35 ± 12.51	NR	NR	280	Placebo
Masoodi et al., 2013 [69]	Iran	Parallel, R, DB, PC	Patients with NAFLD	♂/♀	50	50	12	48.4 ± 6.7	48.32 ± 5.4	29.04 ± 3.6	29.1 ± 3.3	280	Placebo
Ebrahimpourkoujan et al., 2015 [70]	Iran	Parallel, R, TB, PC	Patients with T2DM	♂/♀	20	20	6	43.5 ± 5.7	46.1 ± 4.3	30.7 ± 2.4	30.0 ± 4.4	420	Placebo
Elgarf et al., 2015 [71]	Egypt	Parallel, R, SB, PC	Patients with T2DM	♂/♀	40	20	12	51.5 ± 5.5	51 ± 6	34.2 ± 3.6	34.7 ± 5.2	420	Placebo
Shirali et al., 2016 (A) [72]	Iran	Parallel, R, CO	Non-athletic male	♂	8	8	6	20.3 ± 2.6	20.5 ± 2.2	23.4 ± 4.1	24.2 ± 2.3	500	Endurance training
Shirali et al., 2016 (B) [72]	Iran	Parallel, R, CO	Non-athletic male	♂	8	8	6	20.6 ± 2.8	20.1 ± 2.1	23.6 ± 2.6	2.63 ± 3.1	500	Non-placebo
Shirali et al., 2016 (A) [73]	Iran	Parallel, R, DB, PC	Untrained men	♂	9	9	4	22.8 ± 1.4	22.5 ± 1.5	22.7 ± 2.3	23.5 ± 3.5	140	Placebo + Endurance training
Shirali et al., 2016 (B) [73]	Iran	Parallel, R, DB, PC	Untrained men	♂	9	9	4	22.9 ± 1.2	22.7 ± 1.5	23.8 ± 3.6	23.6 ± 3.5	140	Placebo + Strength training
Kheong et al., 2017 [74]	Malaysia	Parallel, R, DB, PC	Patients with NASH	♂/♀	49	50	48	49.6 ± 12.7	50.1 ± 10.2	30.0 ± 4.0	31.0 ± 4.6	2100	Placebo
Ebrahimpour-koujan et al., 2018 [75]	Iran	Parallel, R, TB, PC	Patients with T2DM	♂/♀	20	20	6	43.5 ± 5.7	46.1 ± 4.3	30.7 ± 2.4	30.0 ± 4.4	420	Placebo
Anushiravani et al., 2019 [76]	Iran	Parallel, R, DB, PC	Patients with NAFLD	♂/♀	30	30	12	NR	NR	25.1 ± 3.7	26.1 ± 3.1	140	Placebo
Ghalandari et al., 2020 (A) [77]	Iran	Parallel, R, CO	Patients with T2DM	♂	15	15	8	47.00 ± 1.7	46.59 ± 1.8	NR	NR	280	Aerobic training
Ghalandari et al., 2020 (B) [77]	Iran	Parallel, R, PC	Patients with T2DM	♂	15	15	8	46.08 ± 1.8	46.58 ± 1.5	NR	NR	280	Placebo
Vidimce et al., 2021 [78]	Australia	Crossover, R, SB, PC	Healthy men	♂	17	17	2	31.8 ± 10.7		24.7 ± 3.29		420	Placebo
Shadmehri et al., 2021 (A) [79]	Iran	Parallel, R, CO	Women with T2DM and obesity	♀	15	15	12	58.8 ± 3.04		32.1 ± 4.8	30.0 ± 8.6	420	Pilates training
Shadmehri et al., 2021 (B) [79]	Iran	Parallel, R, PC	Women with T2DM and obesity	♀	15	15	12	58.8 ± 3.04		32.2 ± 9.06	32.1 ± 2.6	420	Placebo
Memon et al., 2021 [80]	Pakistan	Parallel, PC	Patients with T2DM	♂/♀	100	100	12	49.9 ± 14.5	50.3 ± 13.3	NR	NR	200	OHA
Aryan et al., 2022 [81]	Iran	Parallel, R, DB, PC	Patients with COVID-19	♂/♀	25	25	2	48.76 ± 2.2	49.32 ± 2.2	NR	NR	210	Placebo
Mirhashemi et al., 2022 [82]	Iran	Parallel, R, DB, PC	Patients with NAFLD	♂/♀	27	25	8	37.8 ± 9.9	38.0 ± 10.0	47.2 ± 6.9	48.2 ± 6.9	560	Placebo
Khan et al., 2022 [83]	Pakistan	Parallel, R, PC	Patients with T2DM	♂/♀	30	30	13	50.5	51	34.2	33.7	200	Placebo

Abbreviations: IG, intervention group; CG, control group; DB, double-blinded; SB, single-blinded; TB, triple-blinded; PC, placebo-controlled; CO, controlled; R, randomized; NR, not reported; ♀, female; ♂, male; T2DM, type 2 diabetes mellitus; T1D, type 1 diabetes; NAFLD, non-alcoholic fatty liver disease; NASH, non-alcoholic steatohepatitis; COVID-19, coronavirus disease 2019; MetS, metabolic syndrome; SIL, silymarin; OHA, oral hypoglycemic agents.

**Table 2:** Subgroup analyses of the impacts of silymarin supplementation on cardiovascular risk factors.

	Effect Size, n	WMD (95% CI)	P—within Subgroups	Heterogeneity		
				P—Heterogeneity	I^2^	P between Subgroups
Impacts of SIL on body weight (kg)						
Overall effect	9	−0.69 (−1.69, 0.31)	0.178	0.986	0.0%	
Baseline BMI (kg/m^2^)						
Normal (18.5–24.9	4	−0.72 (−2.87, 1.43)	0.512	0.799	0.0%	0.909
Overweight (25–29.9)	1	0.76 (−5.51, 7.03)	0.812	-	-	
Obese (≥30)	1	−0.60 (−2.91, 1.71)	0.611	-	-	
Trial duration (week)						
≥12	3	−0.79 (−2.77, 1.19)	0.435	0.675	0.0%	0.908
˂12	6	−0.65 (−1.81, 0.50)	0.270	0.961	0.0%	
Intervention dose (mg/day)						
≥400	3	−0.84 (−2.65, 0.97)	0.365	0.650	0.0%	0.846
˂400	6	−0.62 (−1.82, 0.58)	0.310	0.969	0.0%	
Health status						
Unhealthy	5	−0.68 (−1.81, 1.43)	0.239	0.938	0.0%	0.975
Healthy	4	−0.72 (−2.87, 1.43)	0.512	0.799	0.0%	
Impacts of SIL on BMI (kg/m^2^)						
Overall effect	15	−0.36 (−1.28, 0.57)	0.447	<0.001	90.6%	
Baseline BMI (kg/m^2^)						
Normal (18.5–24.9	4	−0.04 (−0.88, 0.79)	0.912	0.955	0.0%	0.052
Overweight (25–29.9)	5	0.16 (−0.30, 0.63)	0.485	0.655	0.0%	
Obese (≥30)	6	−1.27 (−2.33, −0.21)	0.019	0.004	70.9%	
Trial duration (week)						
≥12	9	−0.29 (−1.59, 0.99)	0.650	<0.001	93.3%	0.803
˂12	6	−0.48 (−1.11, 0.14)	0.133	0.742	0.0%	
Intervention dose (mg/day)						
≥400	6	−0.58 (−1.43, 0.26)	0.180	0.930	0.0%	0.689
˂400	9	−0.28 (−1.47, 0.90)	0.639	<0.001	94.1%	
Health status						
Unhealthy	11	−0.45 (−1.53, 0.63)	0.413	<0.001	92.0%	0.562
Healthy	4	−0.04 (−0.88, 0.79)	0.912	0.955	0.0%	
Impacts of SIL on serum FBG (mg/dL)						
Overall effect	24	−21.68 (−31.37, −11.99)	<0.001	<0.001	99.5%	
Baseline serum FBG (mg/dL)						
<126	8	0.16 (−3.28, 3.62)	0.923	0.005	65.8%	<0.001
≥126	16	−33.81 (−44.56, −23.05)	<0.001	<0.001	98.7%	
Baseline BMI (kg/m^2^)						
Normal (18.5–24.9	2	0.11 (−0.28, 0.50)	0.579	0.356	0.0%	0.004
Overweight (25–29.9)	8	−3.02 (−12.07, 6.02)	0.513	<0.001	89.6%	
Obese (≥30)	5	−36.81 (−58.82, −14.80)	0.001	<0.001	98.5%	
Trial duration (week)						
≥12	18	−20.61 (−32.23, −8.99)	0.001	<0.001	98.8%	0.905
˂12	6	−19.65 (−30.28, −9.02)	<0.001	<0.001	97.9%	
Intervention dose (mg/day)						
≥400	14	−25.52 (−39.31, −11.73)	<0.001	<0.001	98.7%	0.404
˂400	10	−17.27 (−30.87, −3.67)	0.013	<0.001	99.2%	
Health status						
Unhealthy	23	−22.62 (−32.10, −13.14)	<0.001	<0.001	98.7%	<0.001
Healthy	1	0.10 (−0.28, 0.49)	0.591	-	-	
Impacts of SIL on serum fasting insulin (mU/mL)						
Overall effect	9	−3.76 (−4.80, −2.72)	<0.001	<0.001	98.6%	
Baseline BMI (kg/m^2^)						
Overweight (25–29.9)	3	−8.53 (−14.08, −2.98)	0.003	<0.001	94.3%	0.655
Obese (≥30)	2	−7.03 (−10.53, −3.53)	<0.001	0.026	79.7%	
Trial duration (week)						
≥12	6	−5.14 (−6.71, −3.56)	<0.001	<0.001	99.1%	<0.001
˂12	3	−1.08 (−1.92, −0.23)	0.012	<0.001	88.0%	
Intervention dose (mg/day)						
≥400	5	−7.06 (−13.27, −0.84)	0.026	<0.001	99.3%	0.049
˂400	4	−0.78 (−1.29, −0.27)	0.003	<0.001	87.1%	
Impacts of SIL on HbA1c (%)						
Overall effect	14	−0.85 (−1.27, −0.43)	<0.001	<0.001	98.4%	
Baseline BMI (kg/m^2^)						
Overweight (25–29.9)	5	−0.31 (−0.67, 0.04)	0.083	<0.001	95.7%	0.151
Obese (≥30)	3	−1.06 (−2.02, −0.11)	0.028	<0.001	97.5%	
Trial duration (week)						
≥12	12	−0.68 (−1.11, −0.24)	0.002	<0.001	98.6%	<0.001
˂12	2	−1.95 (−2.33, −1.57)	<0.001	0.423	0.0%	
Intervention dose (mg/day)						
≥400	9	−0.97 (−1.43, −0.51)	<0.001	<0.001	95.5%	0.396
˂400	5	−0.59 (−1.33, 0.14)	0.117	<0.001	99.1%	
Impacts of SIL on serum TG (mg/dL)						
Overall effect	18	−26.22 (−40.32, −12.12)	<0.001	<0.001	97.1%	
Baseline serum TG (mg/dL)						
<150	3	10.31 (−13.13, 33.76)	0.389	<0.001	95.5%	0.005
≥150	15	−35.30 (−56.99, −13.61)	0.001	<0.001	96.5%	
Baseline BMI (kg/m^2^)						
Normal (18.5–24.9)	1	−4.37 (−4.71, −4.04)	<0.001	-	-	0.049
Overweight (25–29.9)	7	0.91 (−14.32, 16.16)	0.906	<0.001	84.0%	
Obese (≥30)	4	−66.20 (−117.63, −14.76)	0.012	<0.001	96.6%	
Trial duration (week)						
≥12	12	−32.85 (−58.38, −7.32)	0.012	<0.001	97.7%	0.243
˂12	6	−13.37 (−33.80, 7.06)	0.200	<0.001	87.8%	
Intervention dose (mg/day)						
≥400	13	−33.97 (−50.93, −17.02)	<0.001	<0.001	98.0%	0.009
˂400	5	−6.06 (−18.12, 5.99)	0.324	0.427	0.0%	
Health status						
Unhealthy	16	−31.05 (−52.44, −9.67)	0.004	<0.001	97.1%	0.017
Healthy	2	−1.27 (−12.92, 10.37)	0.831	0.186	42.9%	
Impacts of SIL on serum TC (mg/dL)						
Overall effect	19	−13.97 (−23.09, −4.85)	0.003	<0.001	98.3%	
Baseline serum TC (mg/dL)						
<200	11	−8.58 (−16.97, −0.19)	0.045	<0.001	97.0%	0.145
>200	8	−21.02 (−35.51, −6.54)	0.004	<0.001	89.5%	
Baseline BMI (kg/m^2^)						
Normal (18.5–24.9	1	−0.16 (−0.54, 0.22)	0.410	-	-	0.142
Overweight (25–29.9)	8	1.77 (−10.63, 14.17)	0.780	<0.001	95.3%	
Obese (≥30)	4	−28.27 (−56.51, −0.03)	0.050	<0.001	93.9%	
Trial duration (week)						
≥12	13	−11.39 (−27.15, 4.36)	0.157	<0.001	98.6%	0.462
˂12	6	−20.48 (−38.85, −2.10)	0.029	<0.001	91.7%	
Intervention dose (mg/day)						
≥400	14	−18.79 (−29.88, −7.71)	0.001	<0.001	98.7%	0.004
˂400	5	−0.65 (−6.26, 4.96)	0.820	0.625	0.0%	
Health status						
Unhealthy	17	−15.95 (−29.73, −2.17)	0.023	<0.001	98.2%	0.025
Healthy	2	−0.16 (−0.54, 0.22)	0.408	0.926	0.0%	
Impacts of SIL on serum LDL (mg/dL)						
Overall effect	17	−17.13 (−25.63, −8.63)	<0.001	<0.001	97.9%	
Baseline serum LDL (mg/dL)						
<100	9	−5.28 (−9.93, −0.62)	0.026	<0.001	82.5%	0.001
≥100	8	−29.60 (−42.79, −16.41)	<0.001	<0.001	91.6%	
Baseline BMI (kg/m^2^)						
Normal (18.5–24.9	1	−0.13 (−0.41, 0.15)	0.371	-	-	0.014
Overweight (25–29.9)	6	−6.02 (−10.93, −1.10)	0.016	0.064	52.0%	
Obese (≥30)	4	−25.73 (−54.46, 2.99)	0.079	<0.001	95.6%	
Trial duration (week)						
≥12	11	−15.16 (−25.11, −5.21)	0.003	<0.001	95.8%	0.578
˂12	6	−21.43 (−41.16, −1.70)	0.033	<0.001	94.3%	
Intervention dose (mg/day)						
≥400	12	−22.39 (−33.31, −11.46)	<0.001	<0.001	98.5%	0.004
˂400	5	−4.48 (−10.18, 1.21)	0.123	0.175	36.9%	
Health status						
Unhealthy	15	−19.27 (−28.19, −10.36)	<0.001	<0.001	95.0%	<0.001
Healthy	2	−0.13 (−0.41, 0.15)	0.367	0.712	0.0%	
Impacts of SIL on serum HDL (mg/dL)						
Overall effect	17	2.26 (−0.54, 5.06)	0.114	<0.001	97.7%	
Baseline serum HDL (mg/dL)						
<50	9	5.85 (2.74, 8.96)	<0.001	<0.001	95.1%	<0.001
≥50	8	−3.64 (−6.14, −1.14)	0.004	<0.001	78.1%	
Baseline BMI (kg/m^2^)						
Normal (18.5–24.9	1	−4.83 (−4.97, −4.70)	<0.001	-	-	<0.001
Overweight (25–29.9)	6	0.66 (−0.71, 2.04)	0.343	0.068	51.2%	
Obese (≥30)	4	5.30 (−1.19, 11.79)	0.110	<0.001	95.0%	
Trial duration (week)						
≥12	11	0.92 (−2.09, 3.93)	0.549	<0.001	94.5%	0.402
˂12	6	4.65 (−3.55, 12.86)	0.266	<0.001	98.2%	
Intervention dose (mg/day)						
≥400	12	3.11 (−0.43, 6.66)	0.086	<0.001	98.1%	0.368
˂400	5	1.37 (0.05, 2.69)	0.042	0.339	11.7%	
Health status						
Unhealthy	15	2.85 (−0.19, 5.91)	0.067	<0.001	95.3%	0.238
Healthy	2	−1.66 (−8.52, 5.20)	0.635	0.001	90.2%	
Impacts of SIL on serum CRP (mg/L)						
Overall effect	5	−1.10 (−3.13, 0.93)	0.289	<0.001	93.6%	
Baseline serum CRP (mg/L)						
<3	2	−0.22 (−2.66, 2.21)	0.855	<0.001	94.4%	0.075
>3	3	−2.49 (−2.99, −1.98)	<0.001	0.865	0.0%	
Baseline BMI (kg/m^2^)						
Overweight (25–29.9)	1	−3.45 (−13.76, 6.86)	0.512	-	-	0794
Obese (≥30)	2	−2.06 (−3.04, −1.09)	<0.001	0.058	72.3%	
Trial duration (week)						
≥12	2	−2.50 (−3.00, −1.99)	<0.001	0.857	0.0%	0.060
˂12	3	−0.34 (−2.53, 1.85)	0.760	<0.001	89.0%	
Intervention dose (mg/day)						
≥400	3	−1.00 (−3.23, 1.22)	0.379	<0.001	96.8%	0.818
˂400	2	−1.61 (−6.29, 3.07)	0.500	0.695	0.0%	
Health status						
Unhealthy	4	−2.13 (−2.79, −1.47)	<0.001	0.280	21.7%	<0.001
Healthy	1	1.00 (0.29, 1.70)	0.005	-	-	
Impacts of SIL on level of SBP (mmHg)						
Overall effect	3	−1.23 (−2.70, 0.23)	0.099	0.816	0.0%	
Impacts of SIL on level of DBP (mmHg)						
Overall effect	3	−1.25 (−2.25, −0.26)	0.013	0.358	2.6%	

Abbreviations: WMD, weighted mean differences; CI, confidence interval; SIL, silymarin; BMI, body mass index; FBG, fasting blood glucose; HbA1c, hemoglobin A1c; TG, triglycerides; TC, total cholesterol; LDL, low-density lipoprotein; HDL, high-density lipoprotein; DBP, diastolic blood pressure; SBP, systolic blood pressure; CRP, C-reactive protein.

## Supplementary Materials

The following are available at https://www.mdpi.com/article/10.3390/antiox13040390/s1.
