# Supplementary material for: Impacts of Supplementation with Silymarin on Cardiovascular Risk Factors: A Systematic Review and Dose–Response Meta-Analysis
Source: Antioxidants (Basel). 2024 Mar 24;13(4):390. doi: 10.3390/antiox13040390 (PMC11047742; doi:10.3390/antiox13040390)
Supplement: Supplementary file 1 [file antioxidants-13-00390-s001.zip › Supplementary Materials-SIL2-14.03.2024.pdf]

Supplementary Materials

Supplementary Table S1. Risk of bias assessment for included trials in the meta-analysis.

Study	Bias arising from the randomization process (Allocation bias)	Bias due to deviations from the intended interventions (Performance bias)	Bias due to missing outcome data (Attrition bias)	Bias in the measurement of the Outcome (Detection bias)	Bias in the selection of reported results (Reporting bias)	The overall risk of bias
Velussi et al. 1993	H	U	L	L	U	H
Velussi et al. 1997	H	U	L	L	U	H
Simanek et al. 2001	U	L	L	L	L	U
Fallah Huseini et al. 2004	L	L	L	L	U	U
Fallah Huseini et al. 2006	L	L	L	L	U	U
Hussain et al. 2007	U	L	L	L	U	H
Valentova et al. 2008	H	U	L	L	U	H
Ramezani et al. 2008	L	L	L	L	L	L
Hajaghamohammadi et al. 2008	U	U	L	L	U	H
Gharagozloo et al. 2009	U	L	L	L	H	H
Hashemi et al. 2009	U	U	L	L	U	H
Numan et al. 2010	U	L	L	L	U	H
Rezvanian et al. 2011	U	L	L	L	U	H
Fallahzadeh et al.2012	L	L	L	L	L	L
Alkuraishy et al. 2012	H	U	L	L	U	H
Mohammadi et al. 2013	H	U	L	L	U	H
Di Pierro et al. 2013	H	U	L	L	U	H
Taghvaei et al. 2013	L	L	L	L	L	L
Masoodi et al. 2013	U	L	L	L	U	H
Ebrahimpourkoujan et al. 2015	L	L	L	L	L	L
Elgarf et al. 2015	H	U	L	L	U	H
Shirali et al. 2016	H	U	L	L	U	H
Shirali et al. 2016	H	U	L	L	U	H
Kheong et al. 2017	L	L	L	L	L	L
Ebrahimpour-koujan et al. 2018	L	L	L	L	L	L
Anushiravani et al. 2019	L	L	L	L	L	L
Ghalandari et al. 2020	H	U	L	L	U	H
Vidimce et al. 2021	H	U	L	L	U	H
Shadmehri et al. 2021	H	U	L	L	U	H
Momon et al. 2021	H	U	L	L	U	H
Aryan et al. 2022	L	L	L	L	U	U
Mirhashemi et al.2022	U	L	L	L	U	H
Khan et al. 2022	H	U	L	L	U	H

Abbreviations:. L, low risk of bias, H, high risk of bias; U, unclear risk of bias.

Supplementary Table S2. GRADE profile of the impacts of silymarin supplementation on cardiovascular risk factors

Outcomes	Risk of bias	Inconsistency	Indirectness	Imprecision	Publication Bias	Quality of evidence
Body weight	Very serious limitation	No serious limitation	No serious limitation	Serious limitation[2]	No serious limitation	⊕◯◯◯ Very low
BMI	Very serious limitation	Very serious limitation [1]	No serious limitation	Serious limitation[2]	serious limitation [3]	⊕◯◯◯ Very low
FBG	Very serious limitation	Very serious limitation [1]	No serious limitation	No serious limitation	serious limitation [3]	⊕◯◯◯ Very low
Fasting insulin	Very serious limitation	Very serious limitation [1]	No serious limitation	No serious limitation	serious limitation [3]	⊕◯◯◯ Very low
HbA1c	Very serious limitation	Very serious limitation [1]	No serious limitation	No serious limitation	No serious limitation	⊕◯◯◯ Very low
TG	Very serious limitation	Very serious limitation [1]	No serious limitation	No serious limitation	No serious limitation	⊕◯◯◯ Very low
TC	Very serious limitation	Very serious limitation [1]	No serious limitation	No serious limitation	No serious limitation	⊕◯◯◯ Very low
LDL	Very serious limitation	Very serious limitation [1]	No serious limitation	No serious limitation	serious limitation [3]	⊕◯◯◯ Very low
HDL	Very serious limitation	Very serious limitation [1]	No serious limitation	Serious limitation[2]	serious limitation [3]	⊕◯◯◯ Very low
CRP	Very serious limitation	Very serious limitation [1]	No serious limitation	Serious limitation[2]	No serious limitation	⊕◯◯◯ Very low
SBP	Very serious limitation	No serious limitation	No serious limitation	Serious limitation[2]	serious limitation [3]	⊕◯◯◯ Very low
DBP	Very serious limitation	No serious limitation	No serious limitation	No serious limitation	No serious limitation	⊕⊕◯◯ Low

Abbreviations: BMI, body mass index; FBG, fasting blood glucose; HbA1c, hemoglobin A1c; TG. Triglycerides; TC, total cholesterol; LDL, low-density lipoprotein; HDL, high-density lipoprotein; DBP, diastolic blood pressure; SBP, systolic blood pressure; CRP, C-reactive protein

1- There is significant heterogeneity.
2- There is no evidence of significant effects of silymarin consumption.

(A)

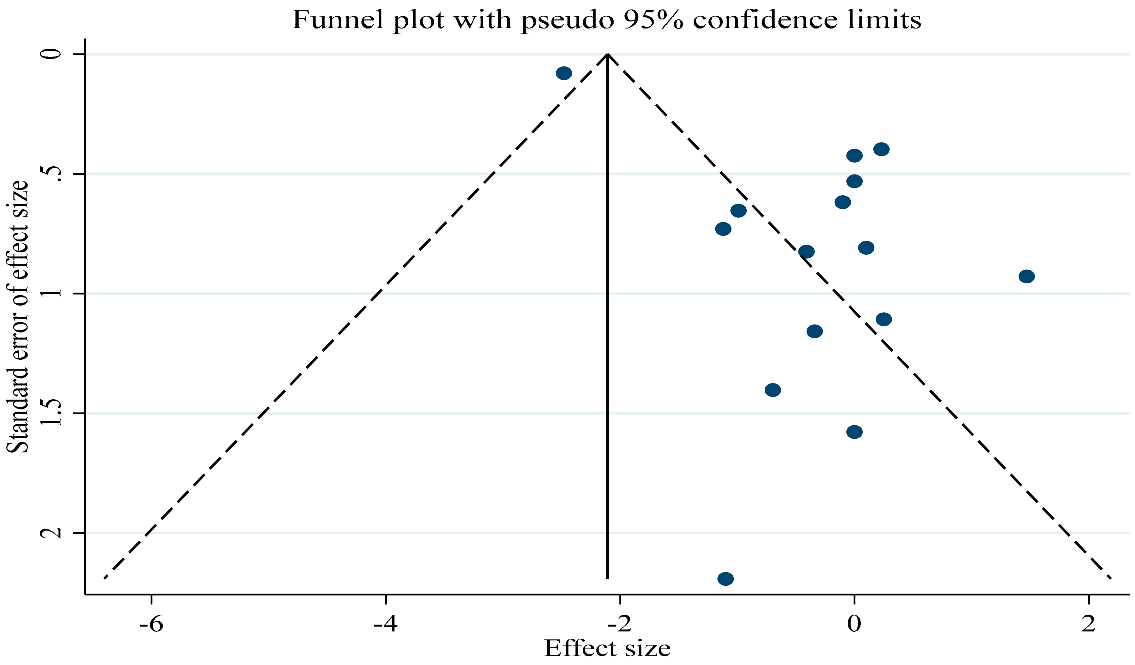

(B)

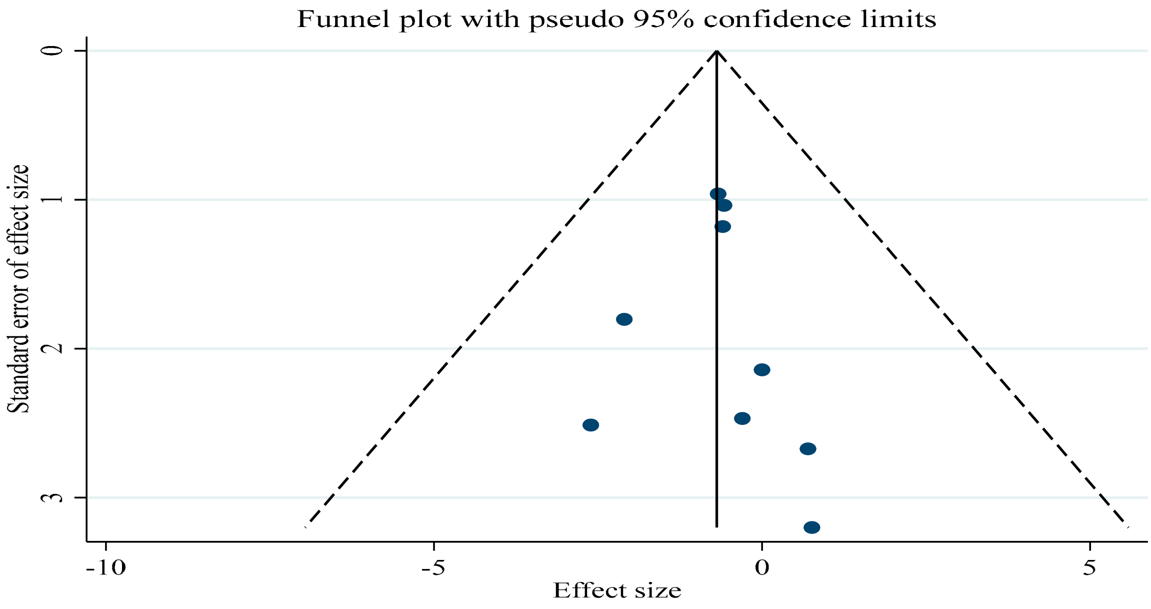

Supplementary Figure S1. *Cont.*

(C)

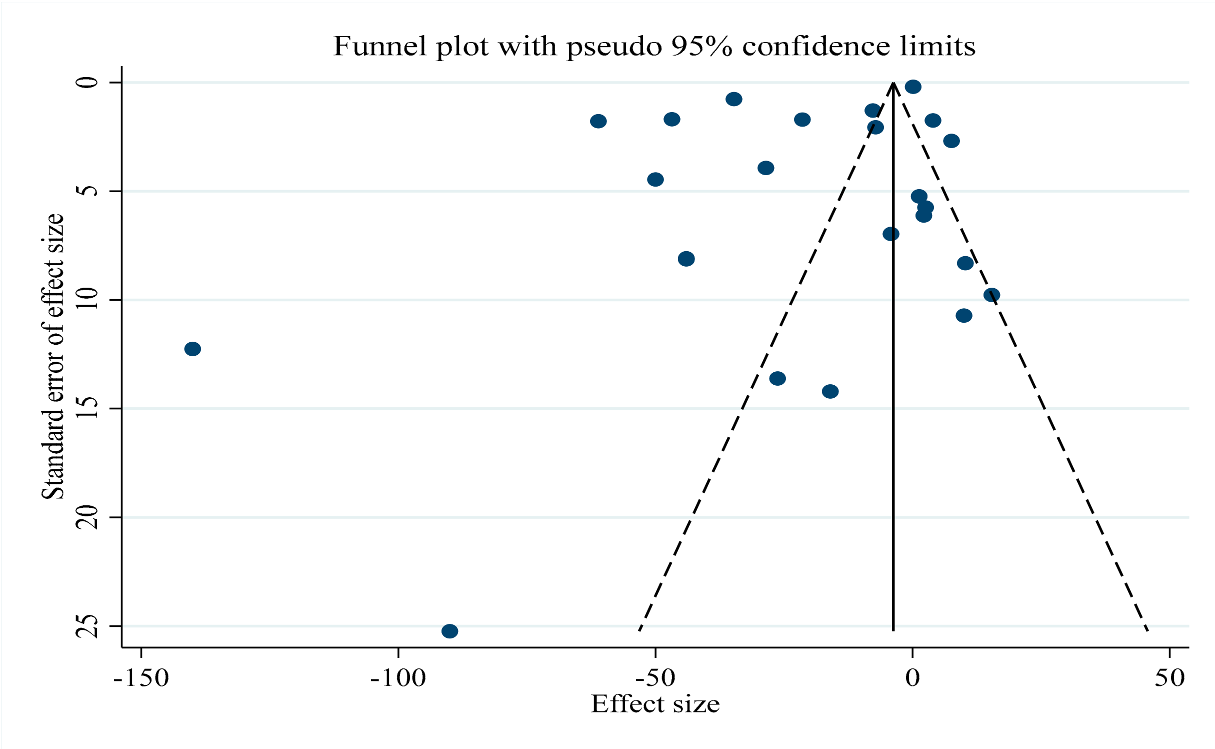

(D)

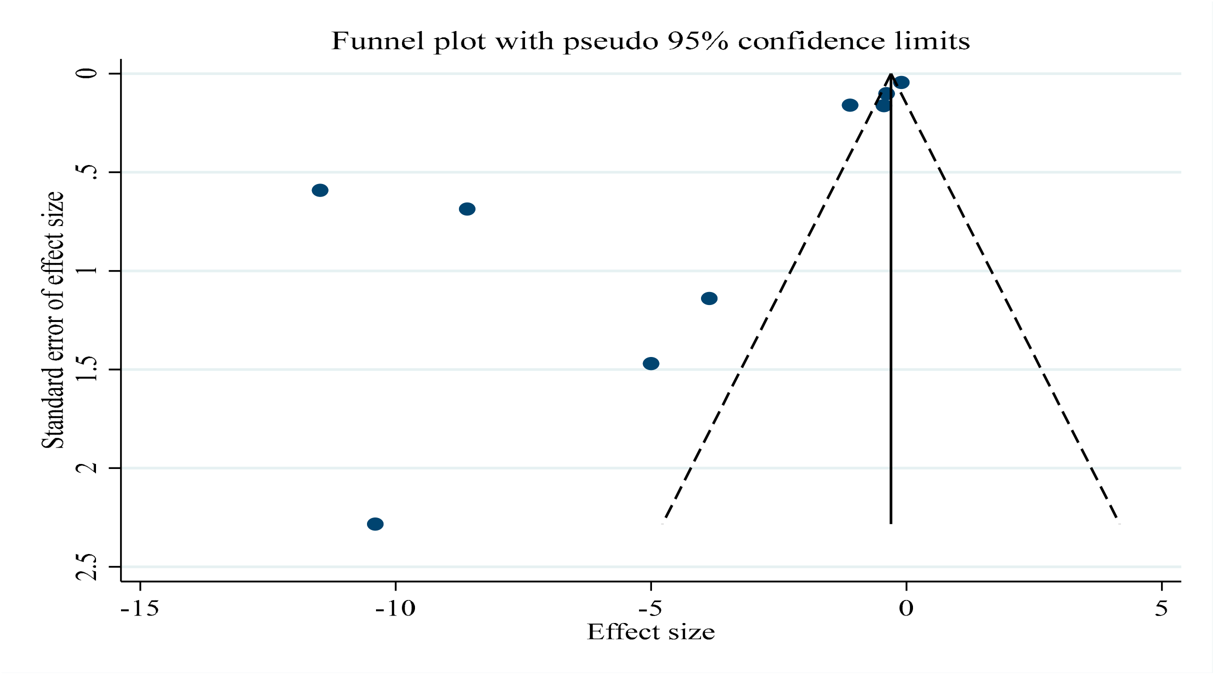

Supplementary Figure S1. *Cont.*

(E)

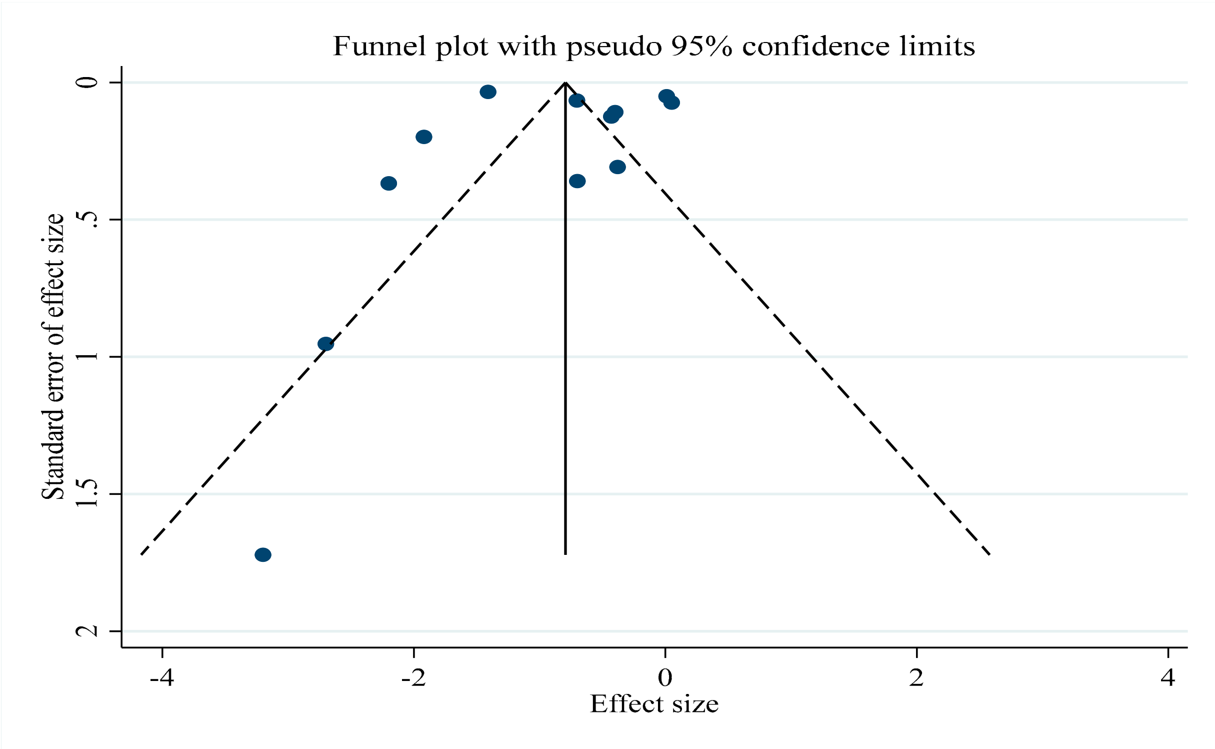

(F)

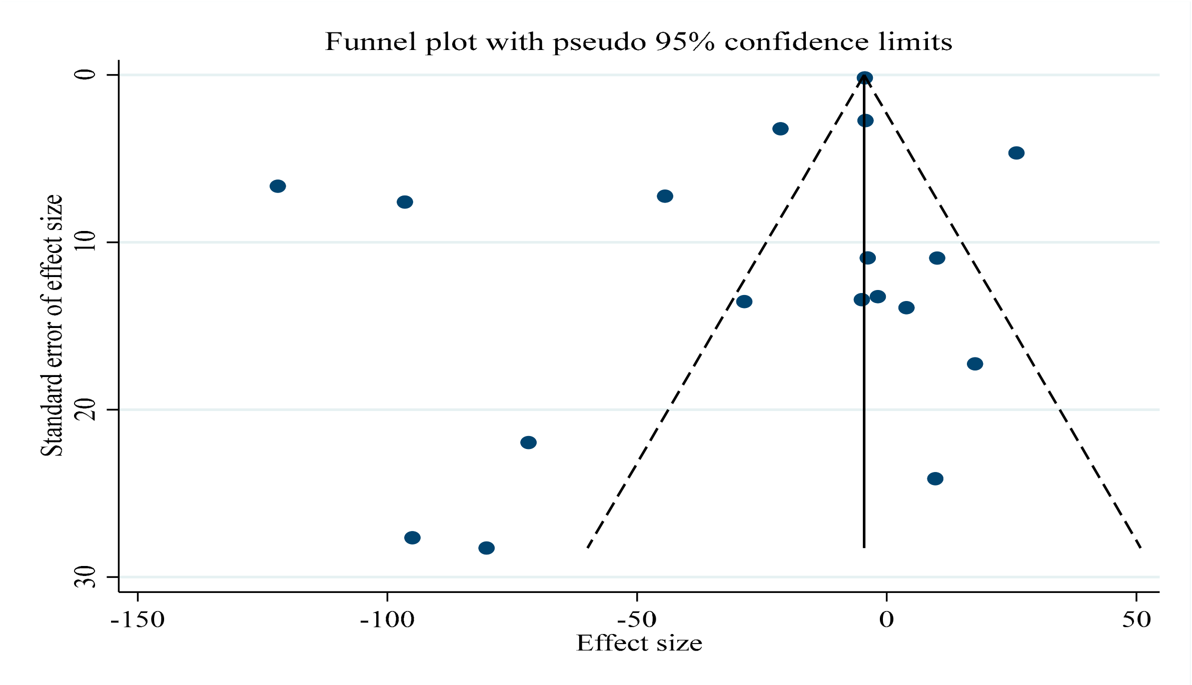

Supplementary Figure S1. *Cont.*

(G)

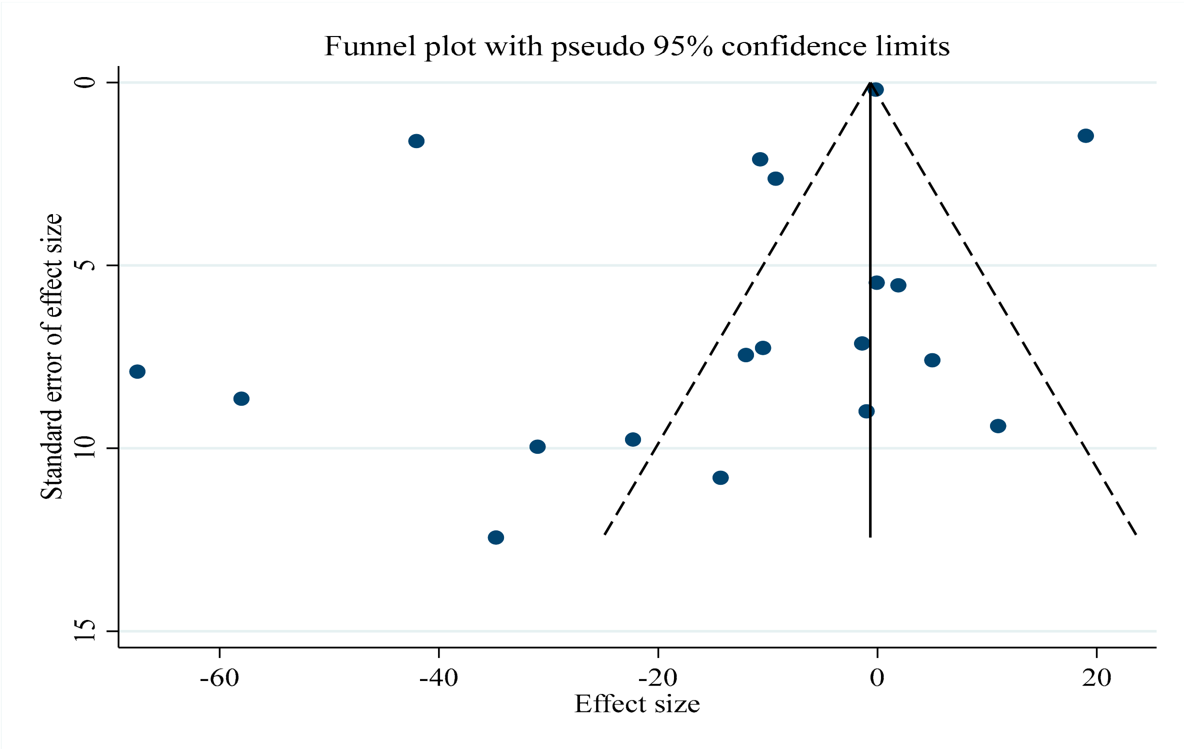

(H)

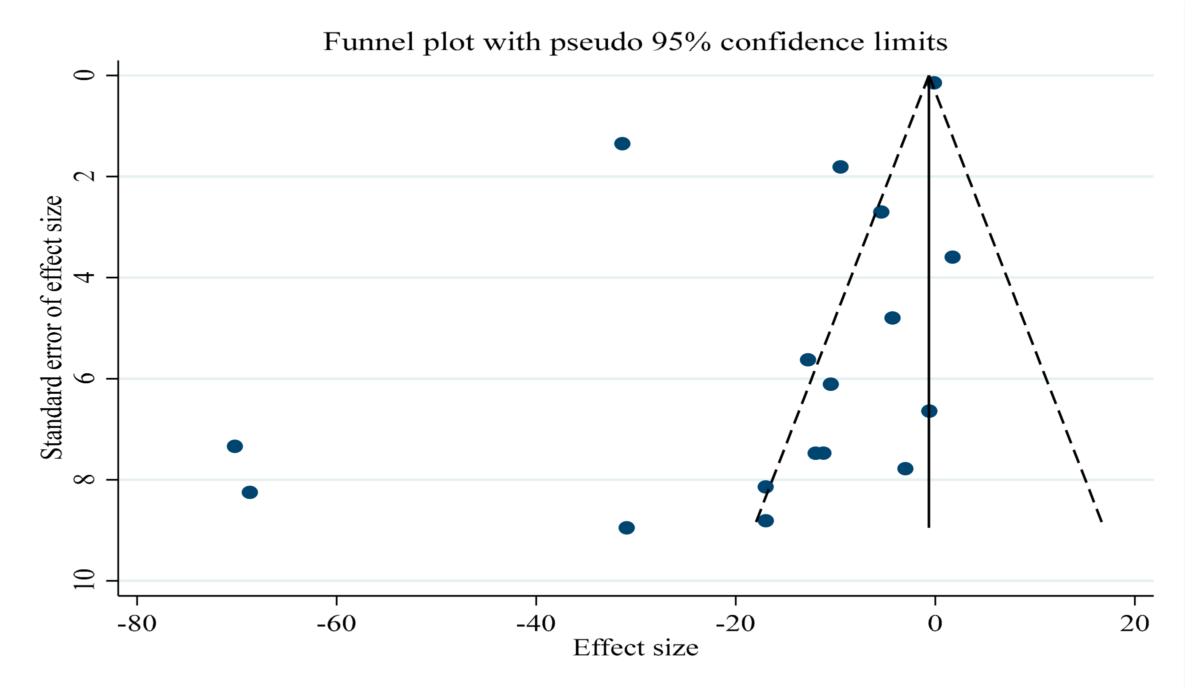

Supplementary Figure S1. *Cont.*

(I)

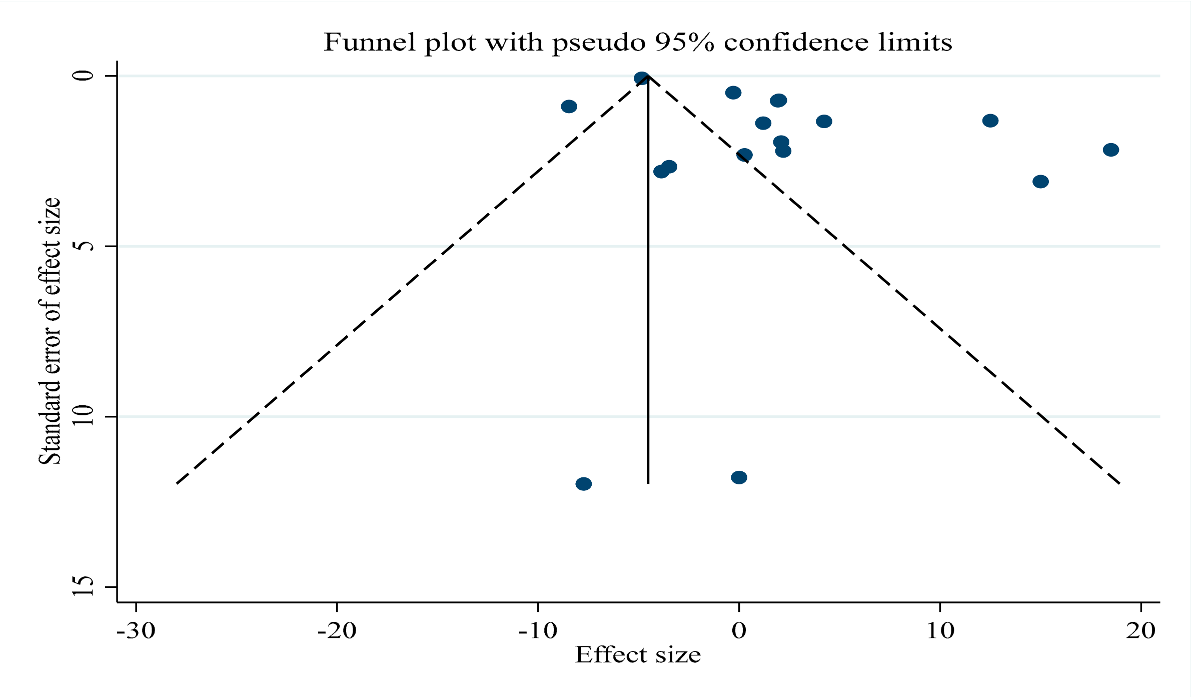

(J)

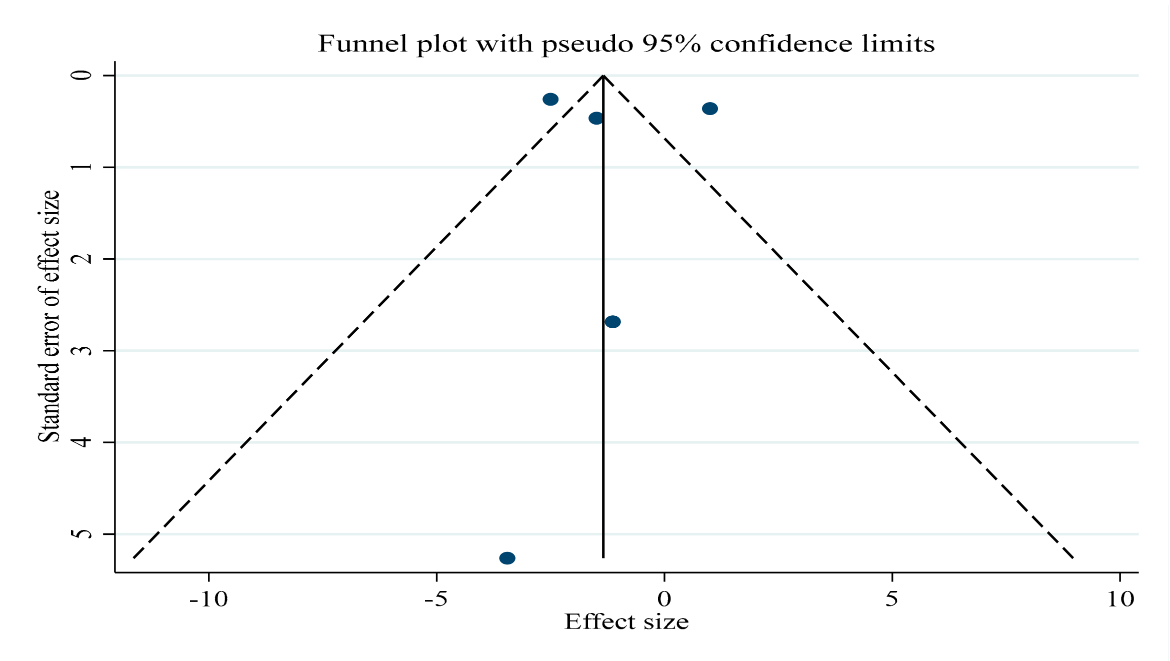

Supplementary Figure S1. *Cont.*

(K)

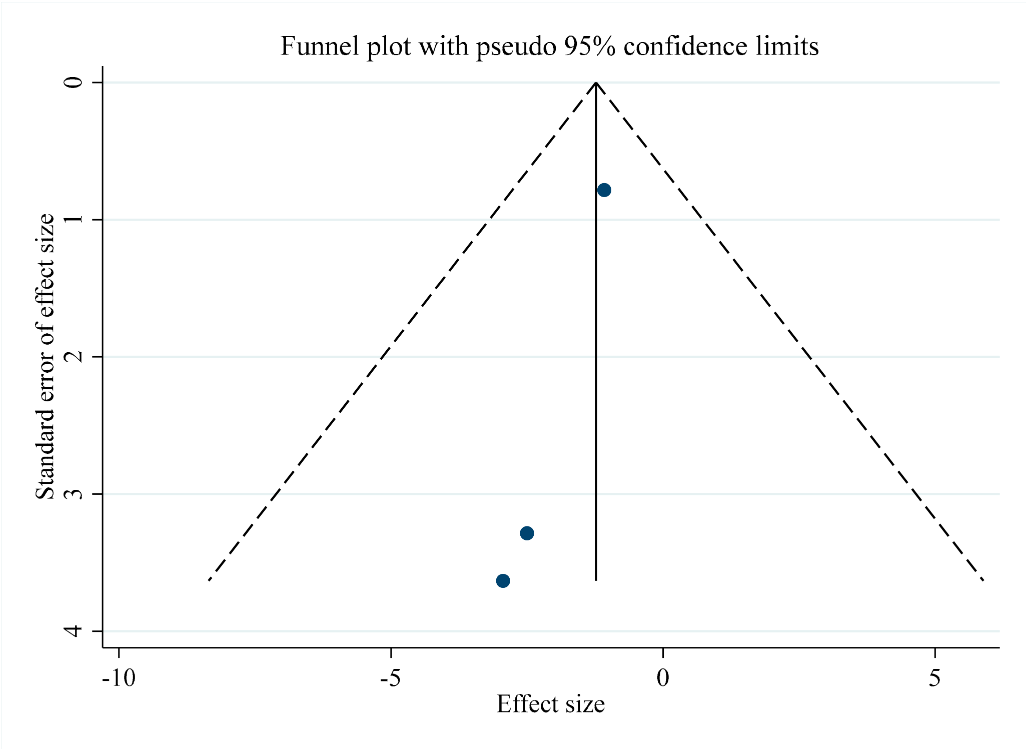

(L)

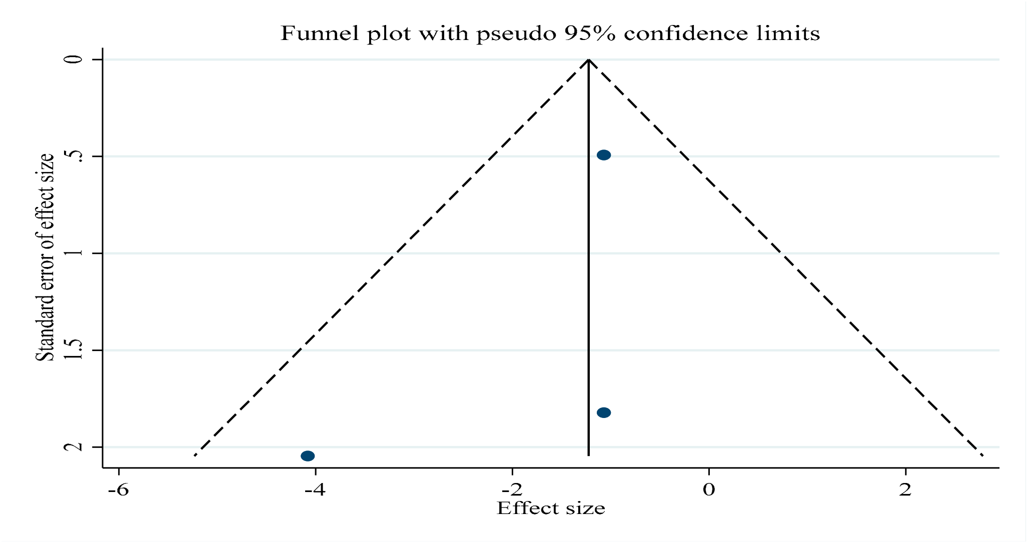

Supplementary Figure S1. Funnel plots for the effect of silymarin supplementation on **A)** BMI (kg/m^2); **B)** body weight (kg); **C)** FBG (mg/dL), **D)** fasting insulin (mU/ml), **E)** HbA1c (%), **F)** TG (mg/dL), **G)** TC (mg/dL), **H)** LDL (mg/dL), **I)** HDL (mg/dL), **J)** CRP (mg/dL), **K)** SBP (mmHg), and **L)** SBP (mmHg).

(A)

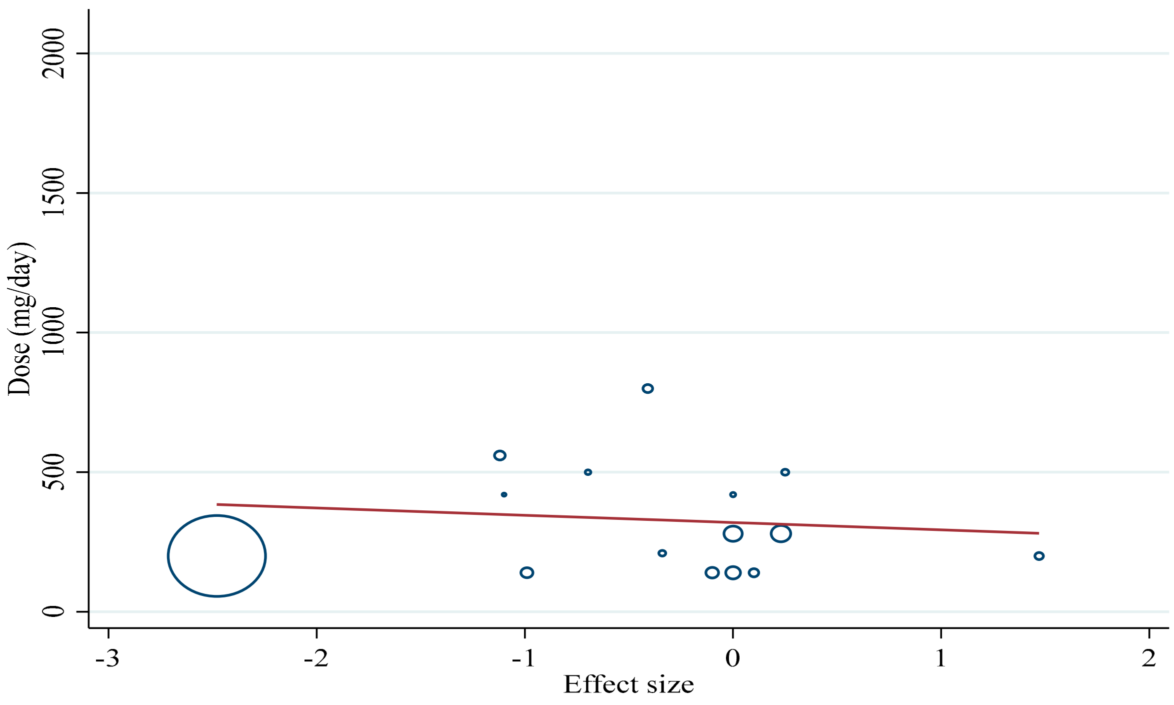

(B)

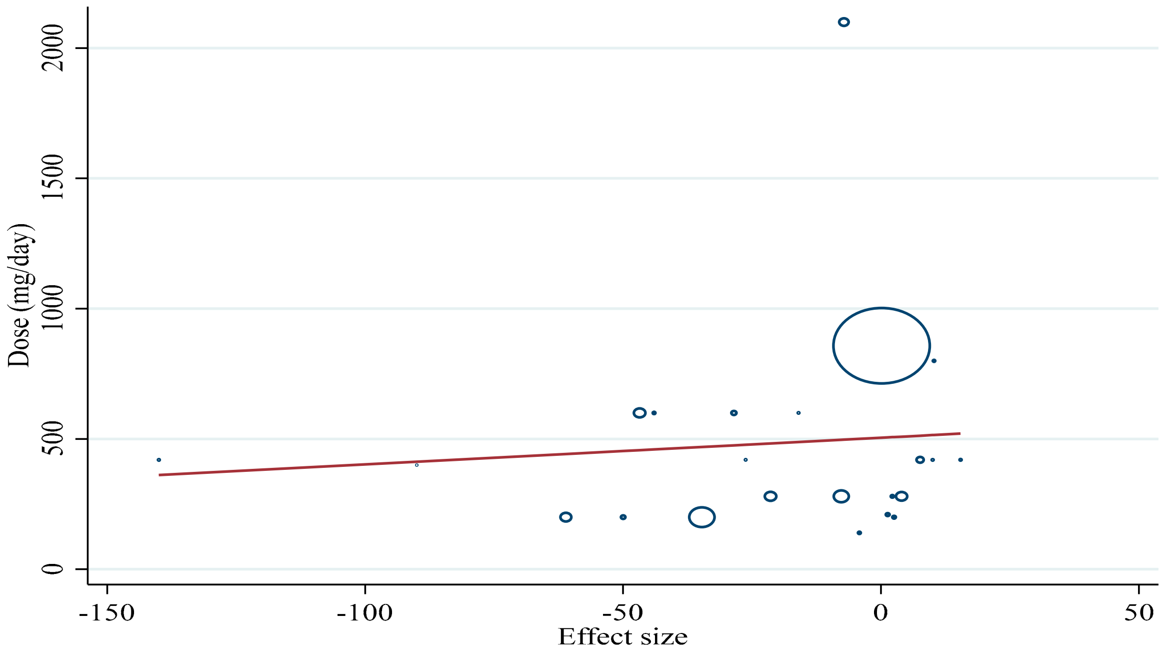

Supplementary Figure S2. *Cont.*

(C)

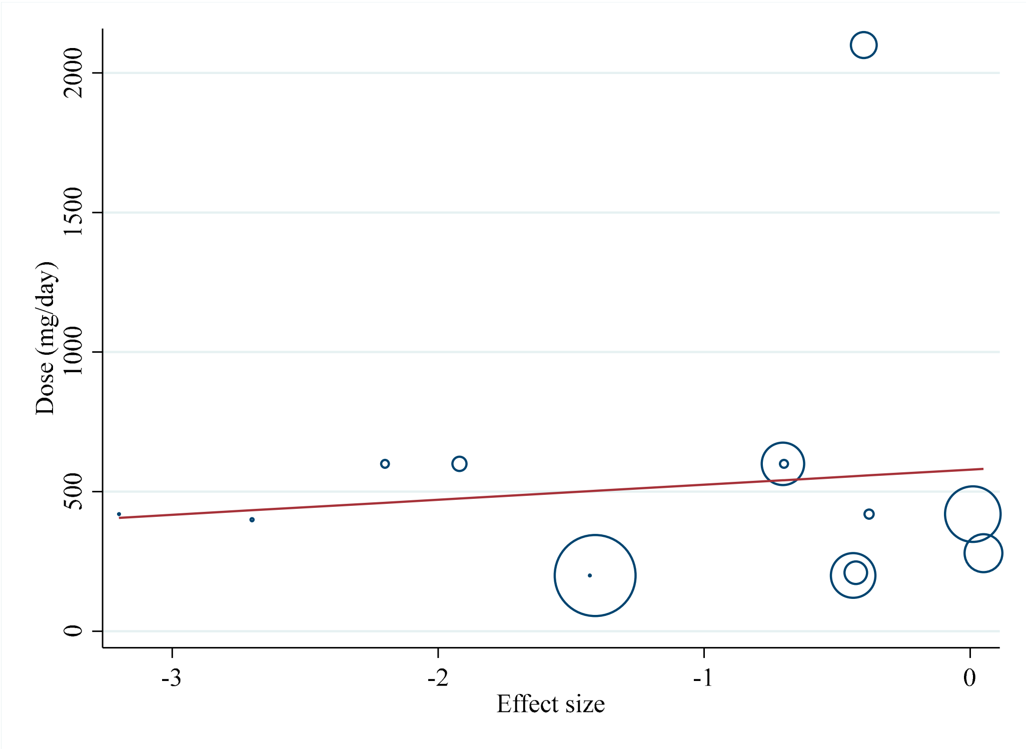

(D)

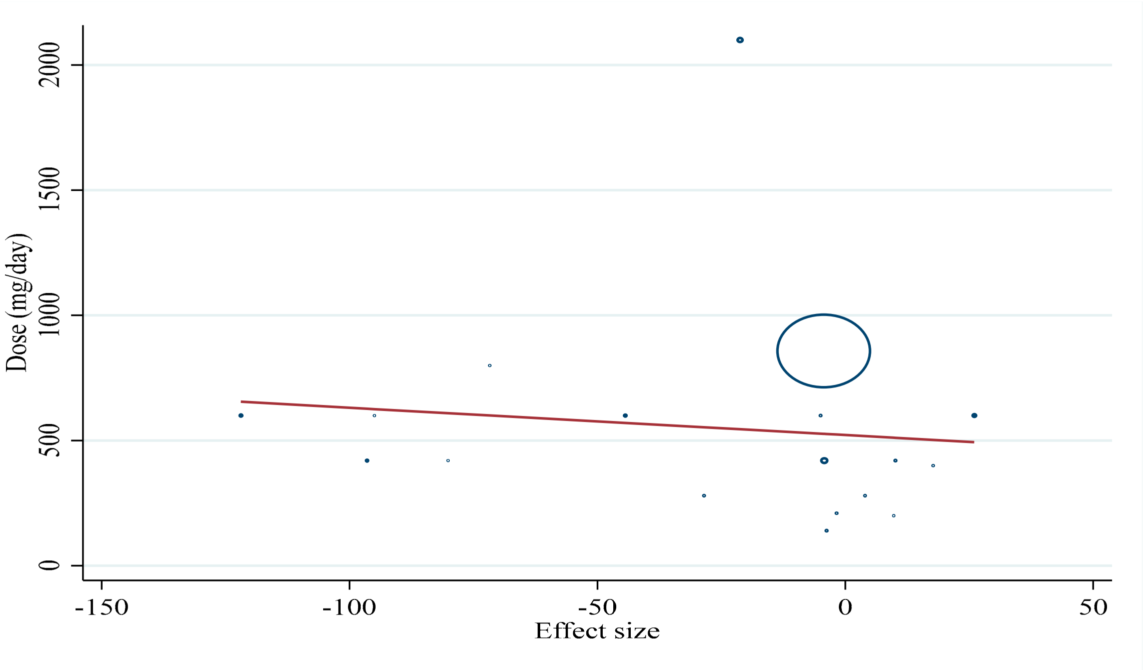

Supplementary Figure S2. *Cont.*

(E)

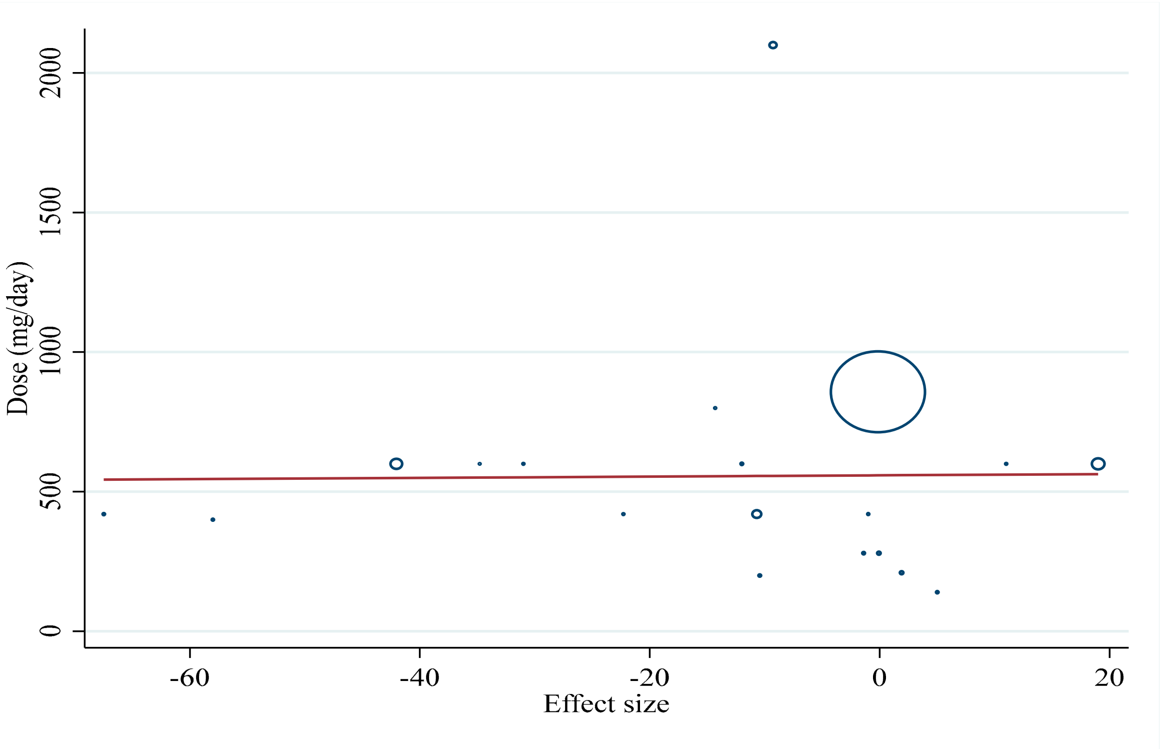

(F)

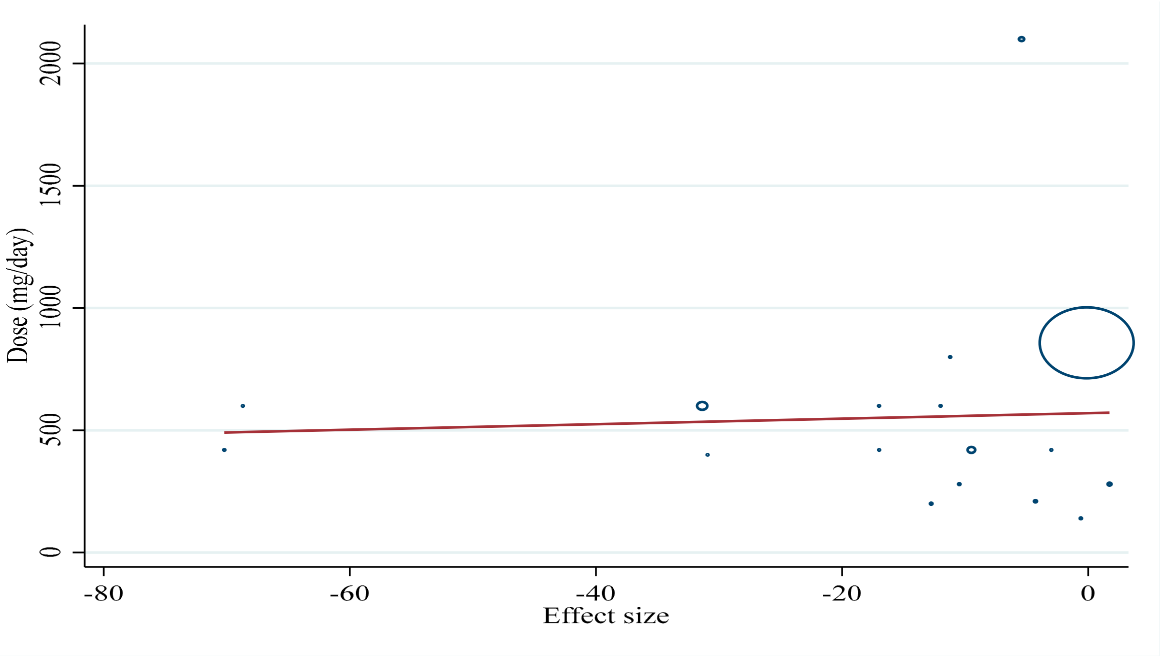

Supplementary Figure S2. *Cont.*

(G)

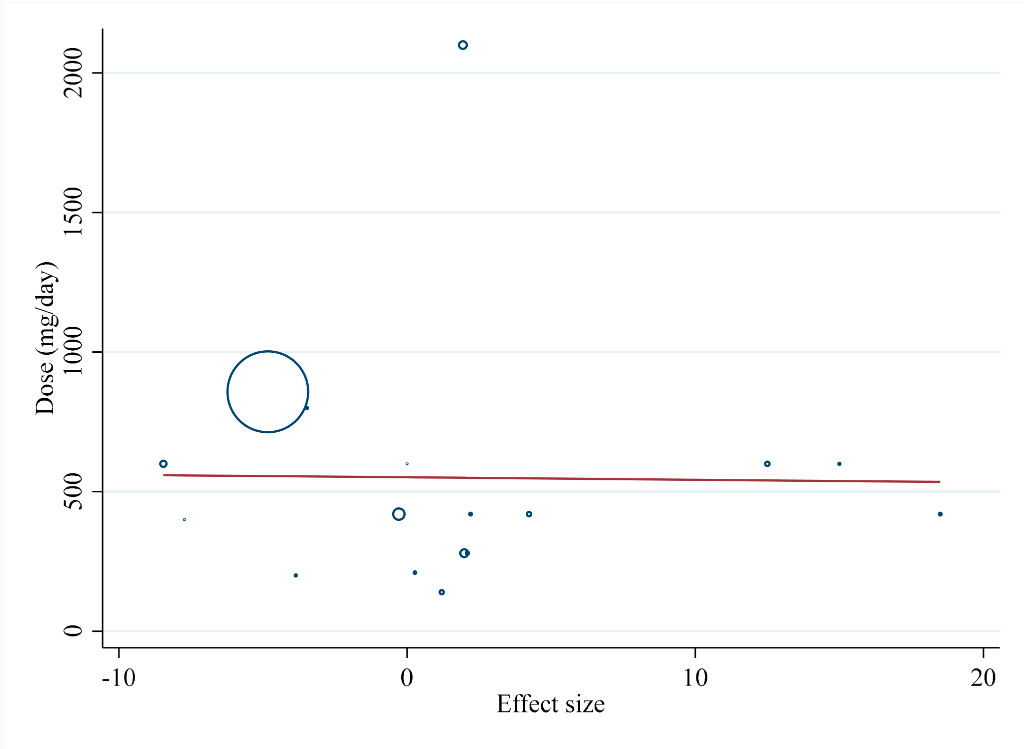

Supplementary Figure S2. Non-linear dose-response association between dose (mg/day) of silymarin supplementation and absolute mean differences in **A)** BMI (kg/m^2), **B)** FBG (mg/dL), **C)** HbA1c (%), **D)** TG (mg/dL), **E)** TC (mg/dL), **F)** LDL (mg/dL), and **G)** HDL (mg/dL).

(A)

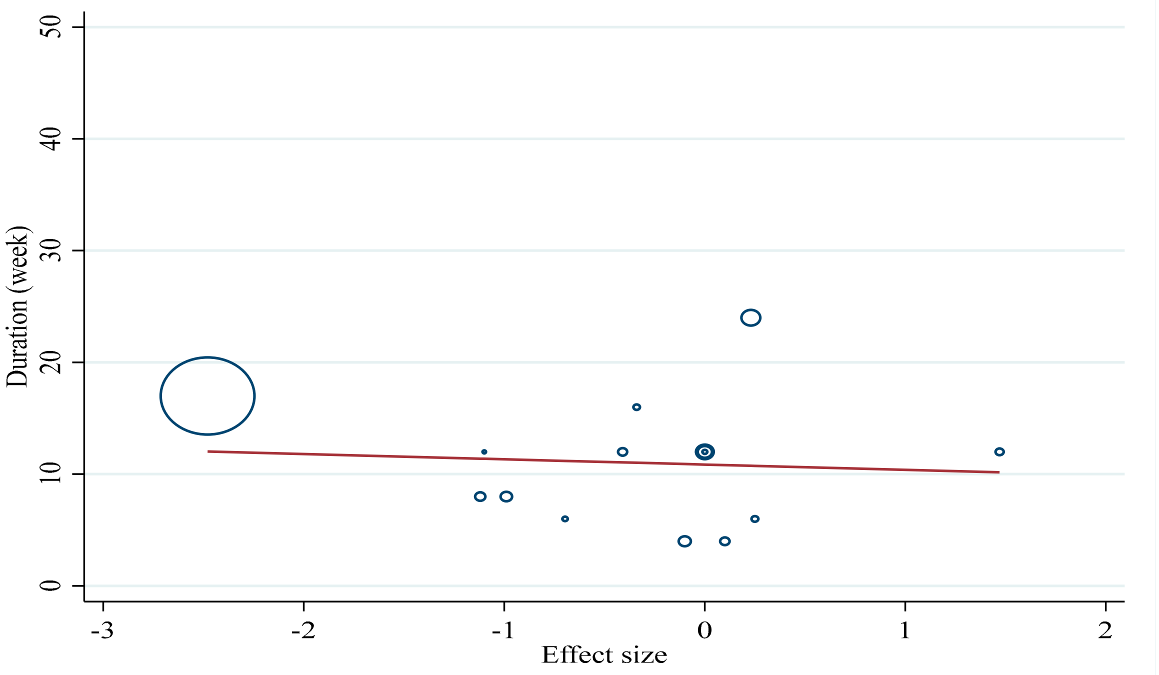

(B)

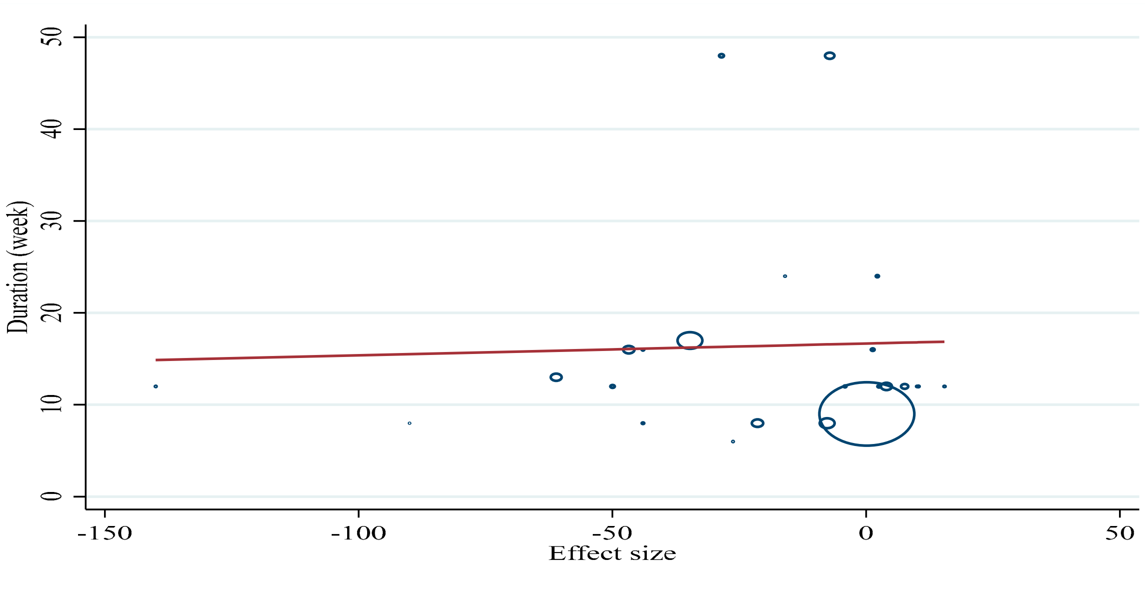

Supplementary Figure S3. *Cont.*

(C)

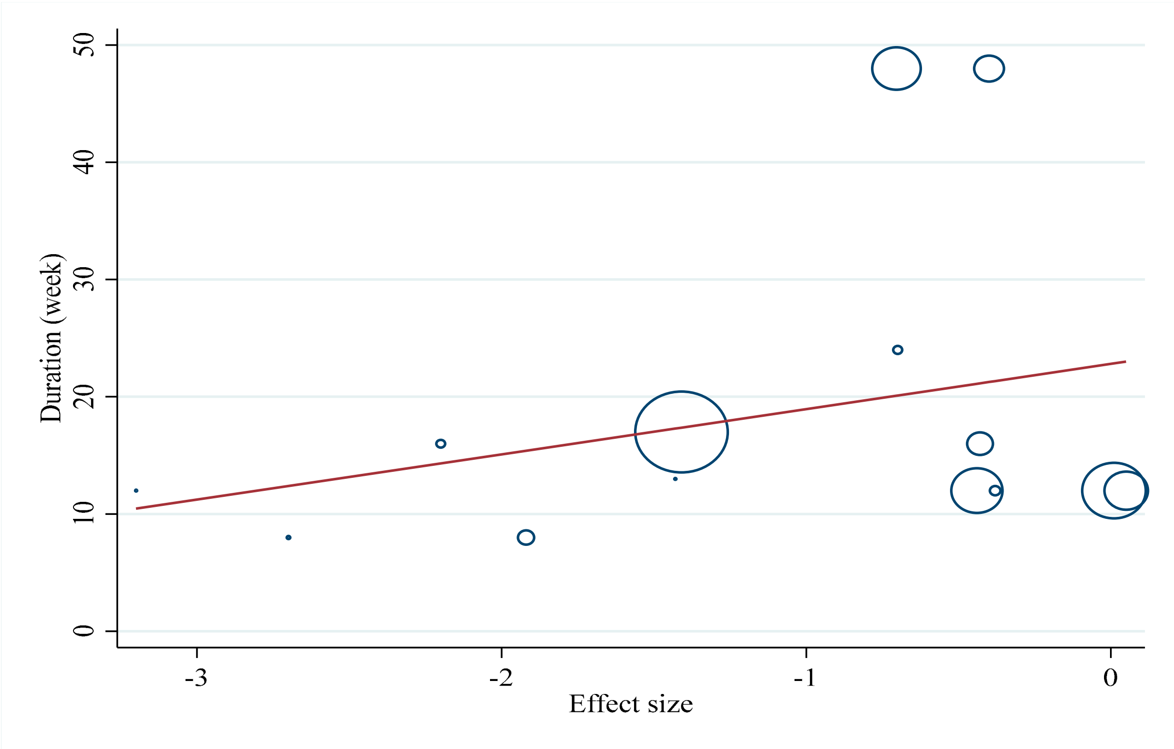

(D)

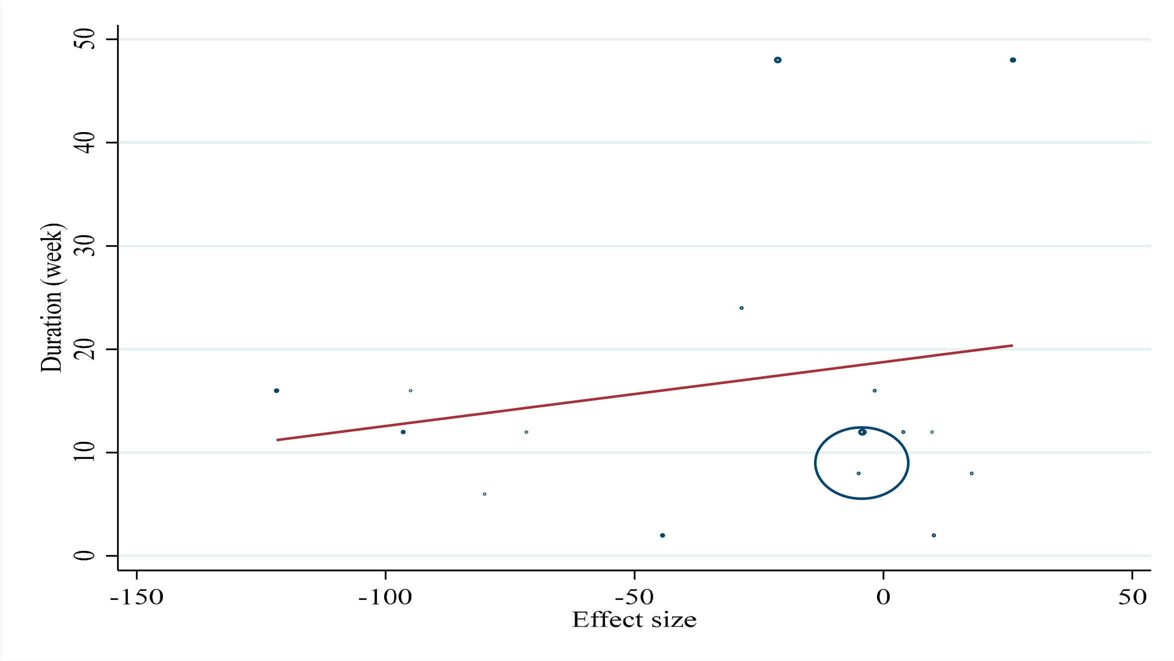

Supplementary Figure S3. *Cont.*

(E)

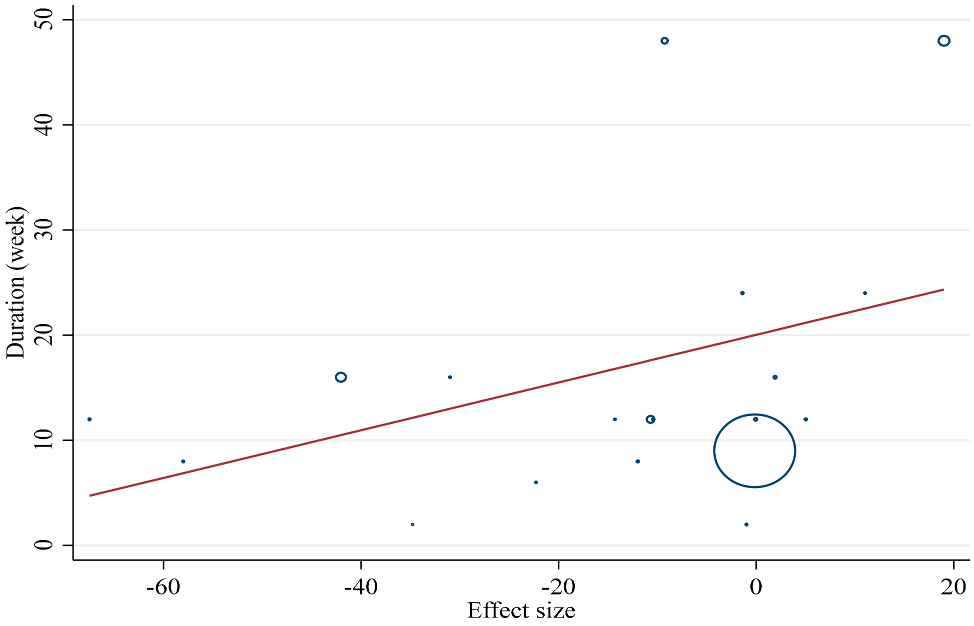

(F)

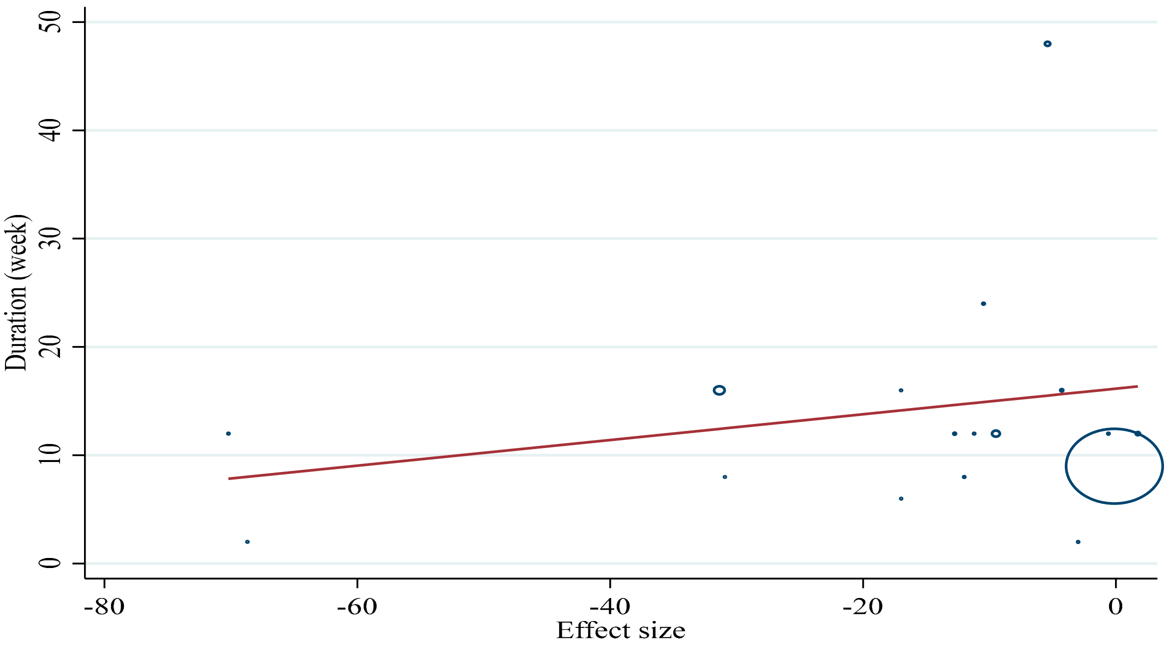

Supplementary Figure S3. *Cont.*

(G)

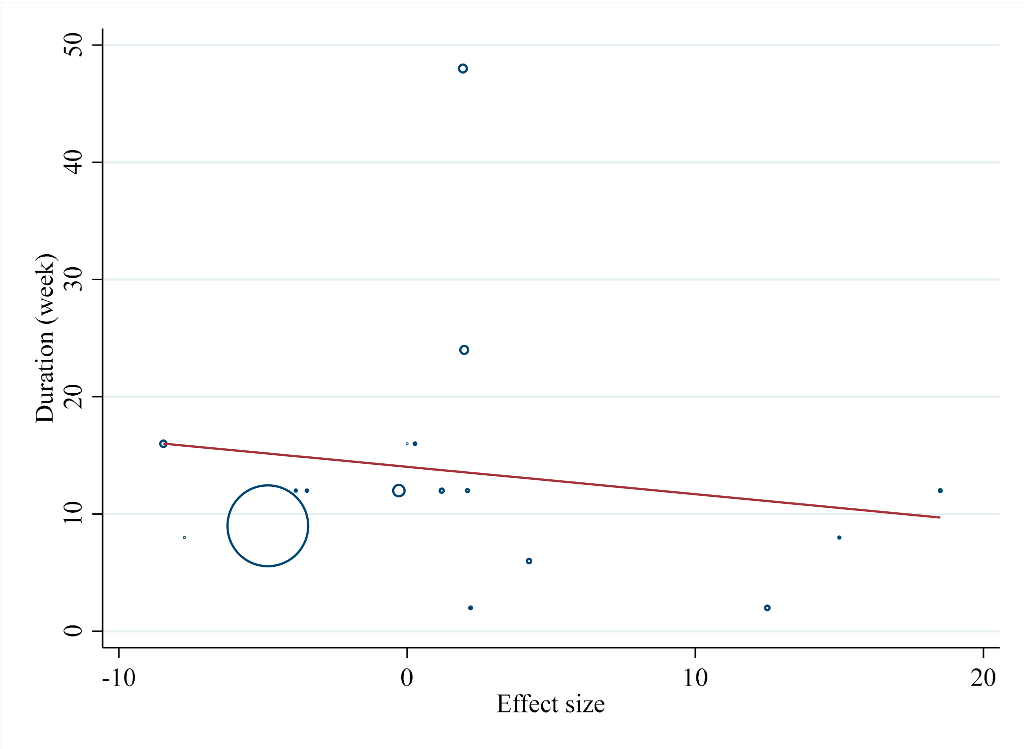

Supplementary Figure S3. Non-linear association between duration (week) of the supplementation with silymarin and absolute mean differences in **A)** BMI (kg/m^2), **B)** FBG (mg/dL), **C)** HbA1c (%), **D)** TG (mg/dL), **E)** TC (mg/dL), **F)** LDL (mg/dL), and **G)** HDL (mg/dL).

(A)

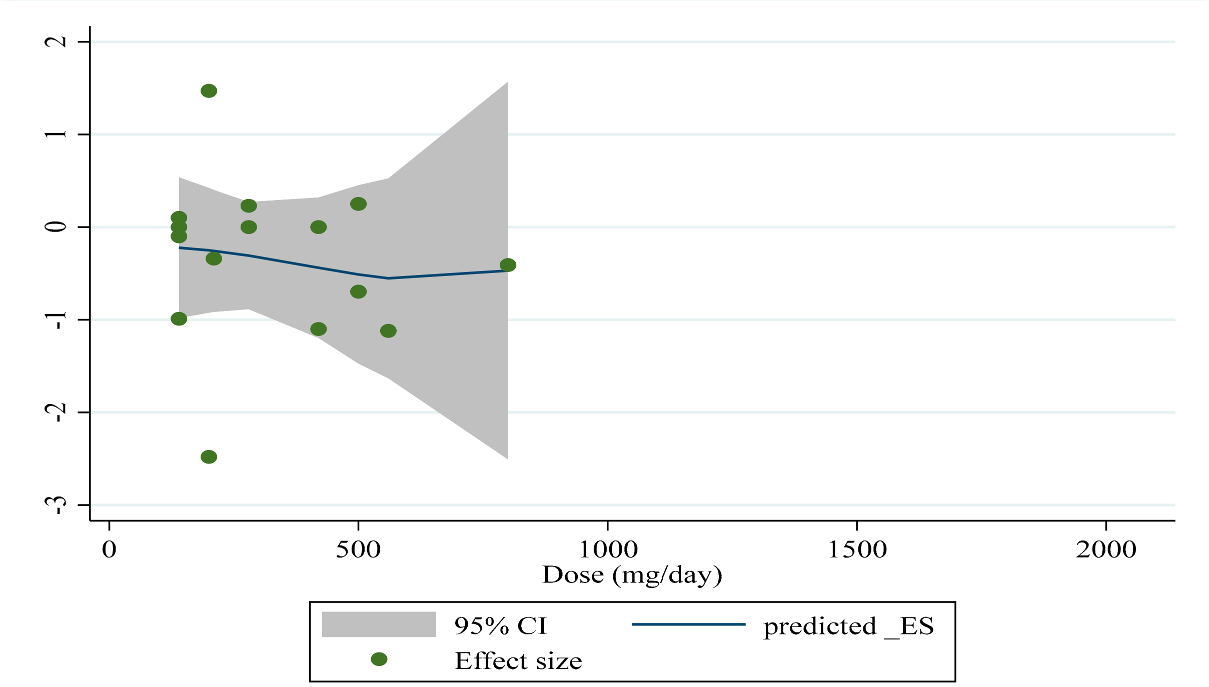

(B)

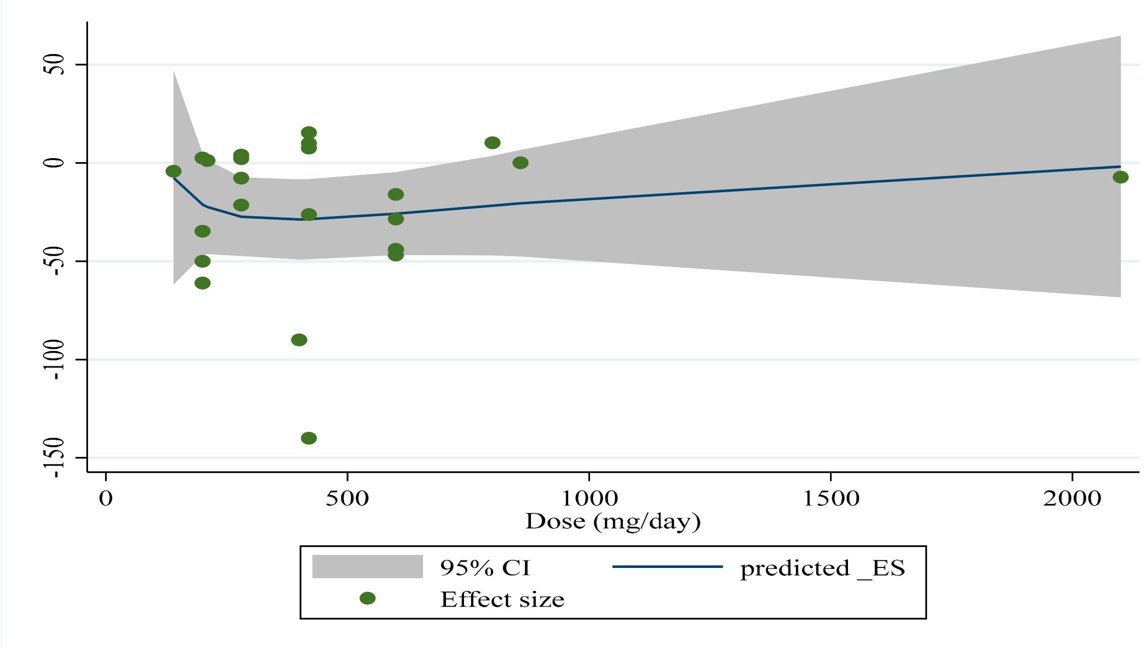

Supplementary Figure S4. *Cont.*

(C)

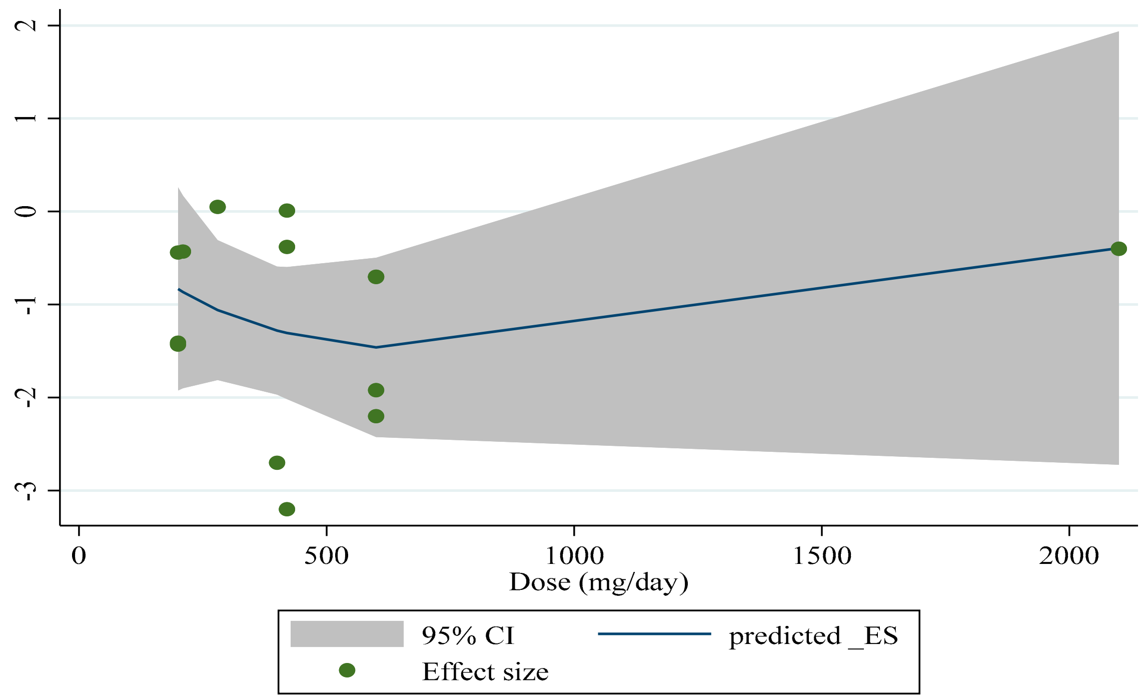

(D)

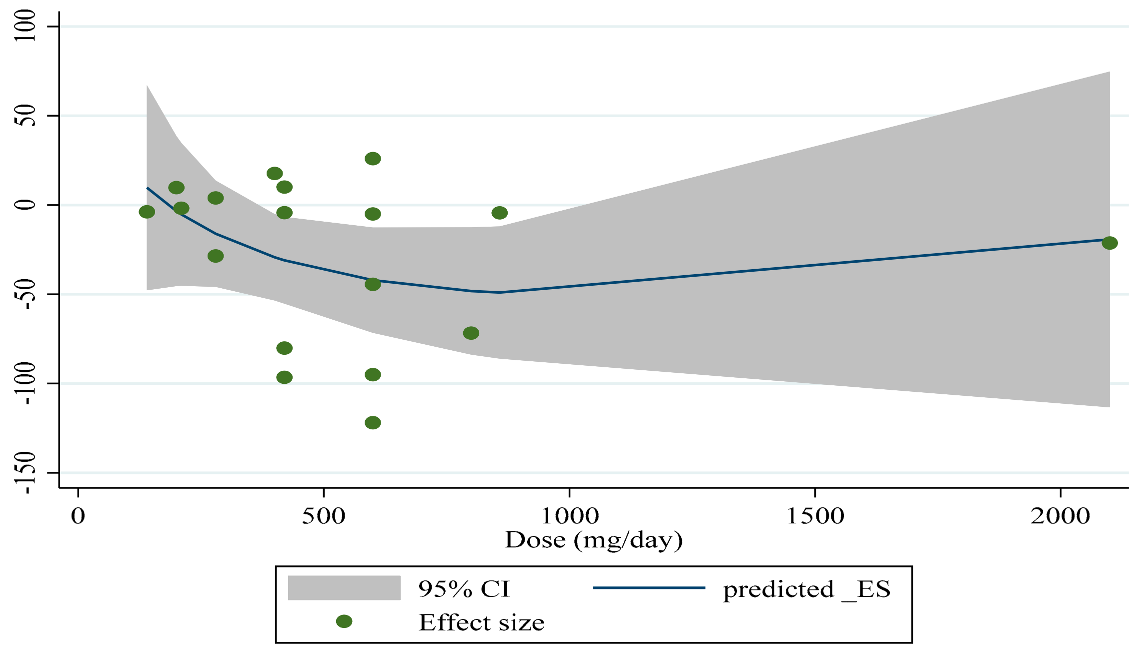

Supplementary Figure S4. *Cont.*

(E)

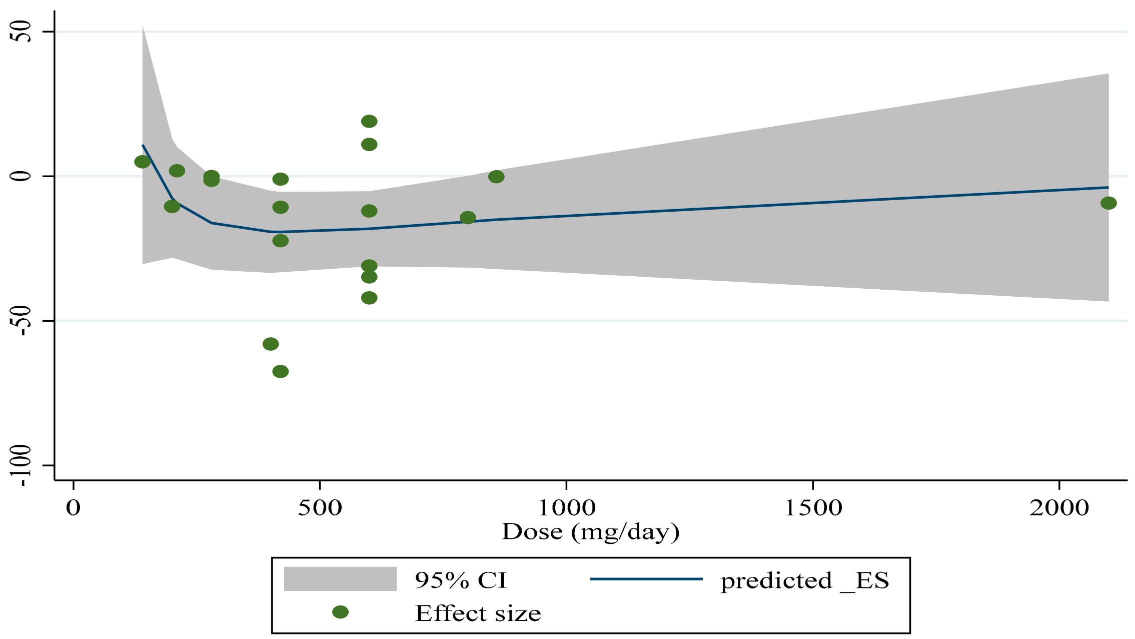

(F)

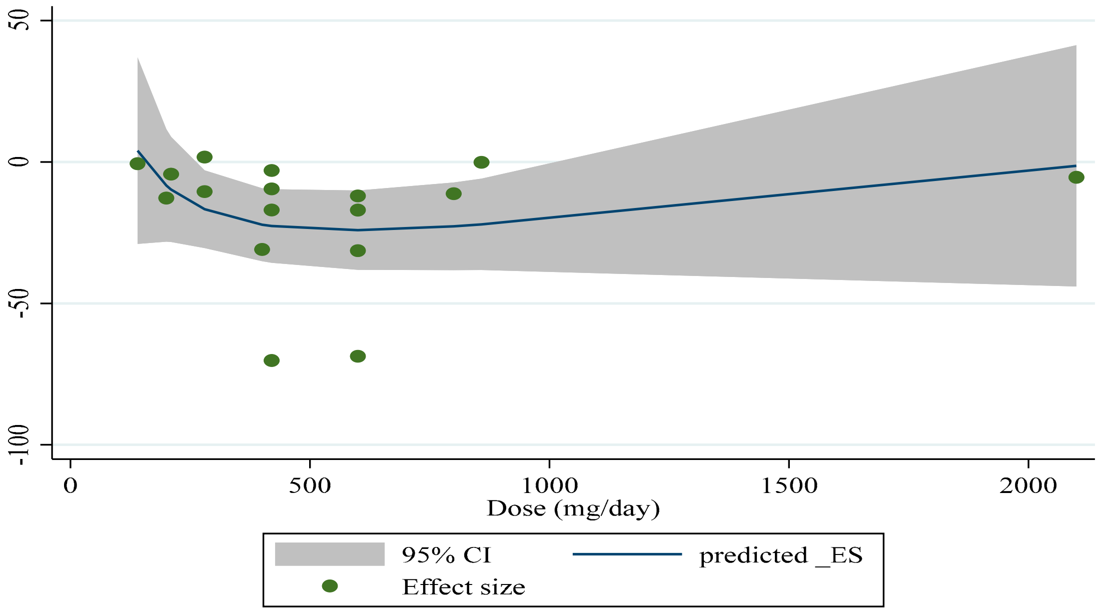

Supplementary Figure S4. *Cont.*

(G)

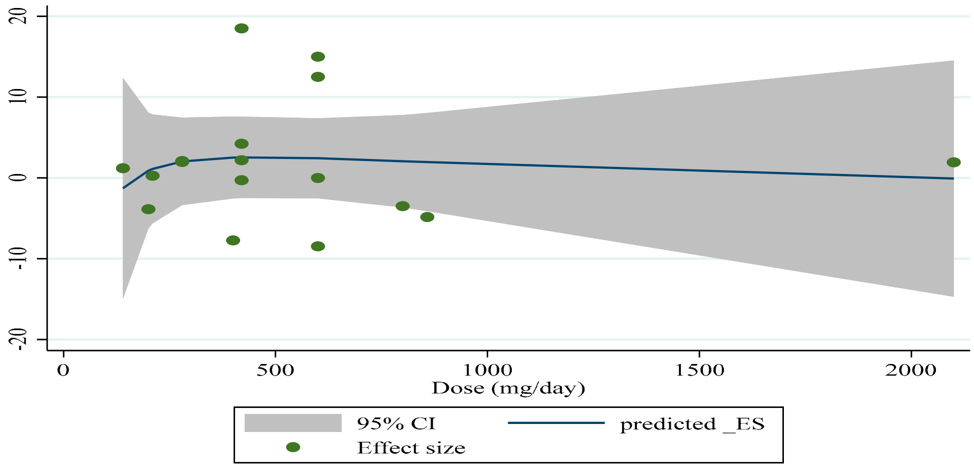

Supplementary Figure S4. Linear dose-response association between dose (mg/day) of silymarin supplementation and absolute mean differences in **A)** BMI (kg/m^2), **B)** FBG (mg/dL), **C)** HbA1c (%), **D)** TG (mg/dL), **E)** TC (mg/dL), **F)** LDL (mg/dL), and **G)** HDL (mg/dL).

(A)

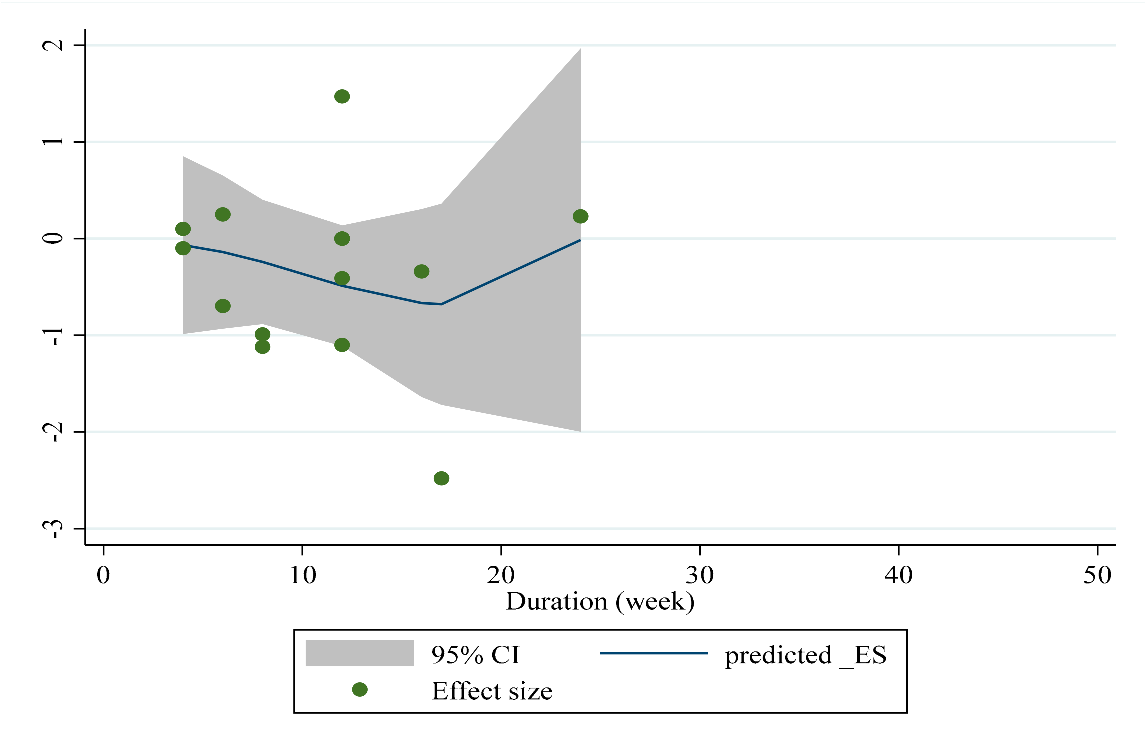

(B)

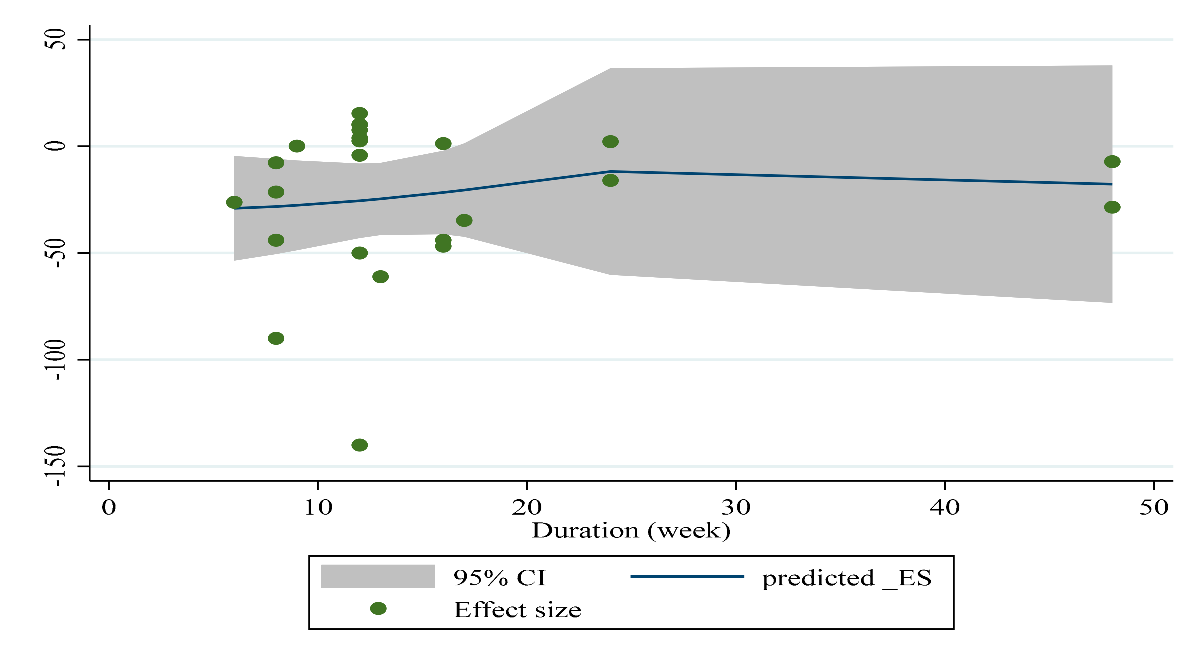

Supplementary Figure S5. *Cont.*

(C)

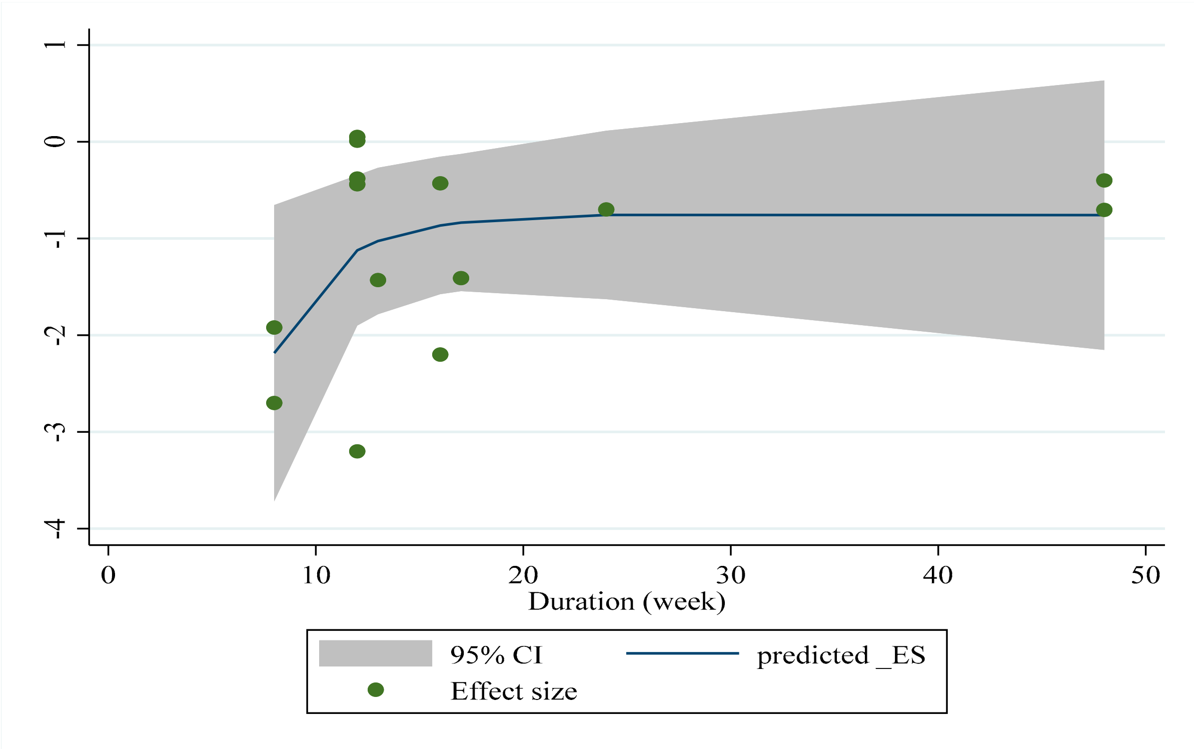

(D)

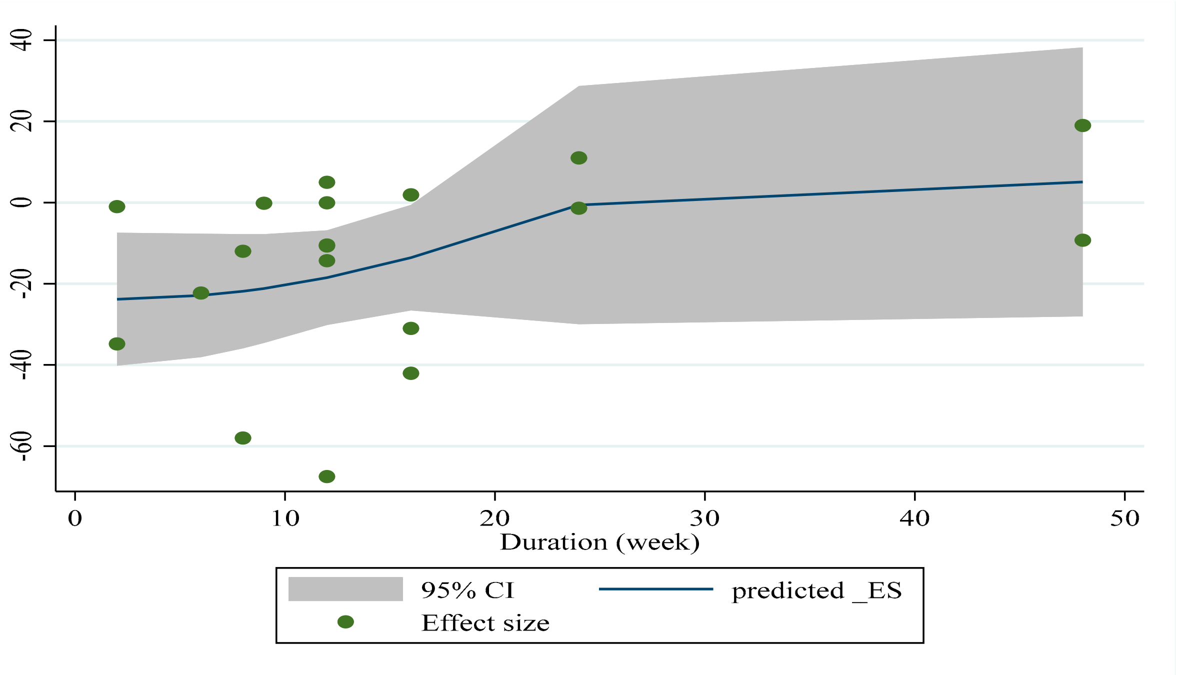

Supplementary Figure S5. *Cont.*

(E)

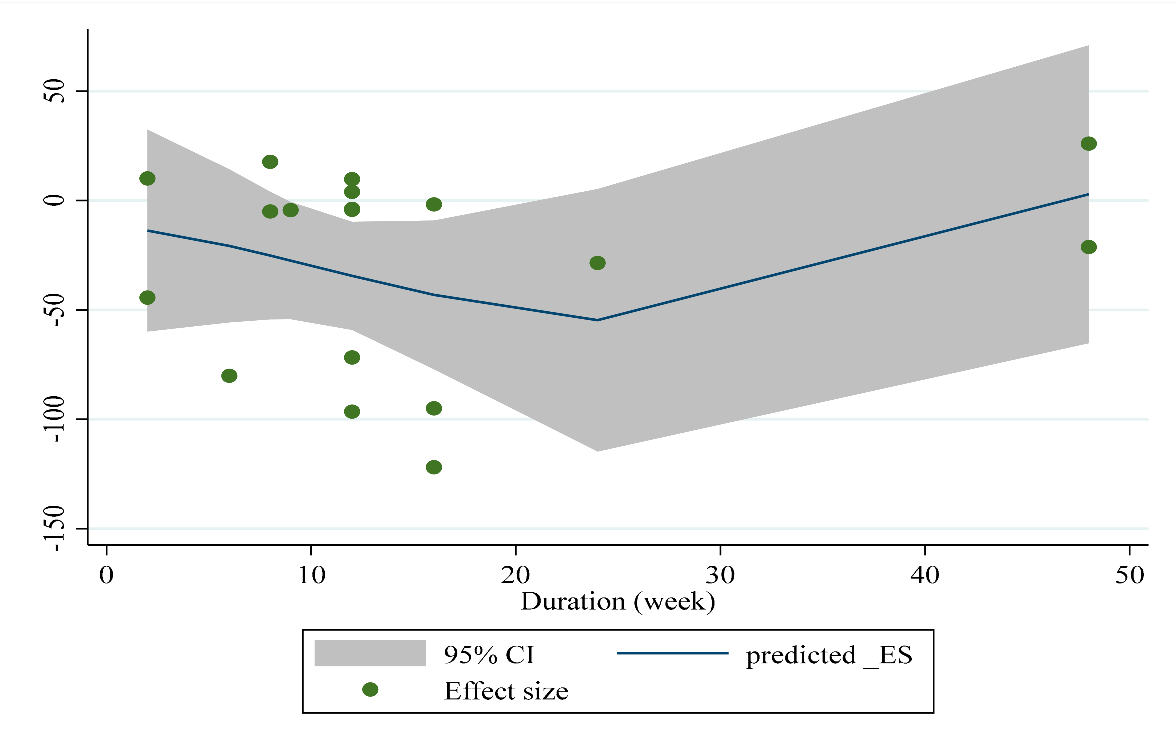

(F)

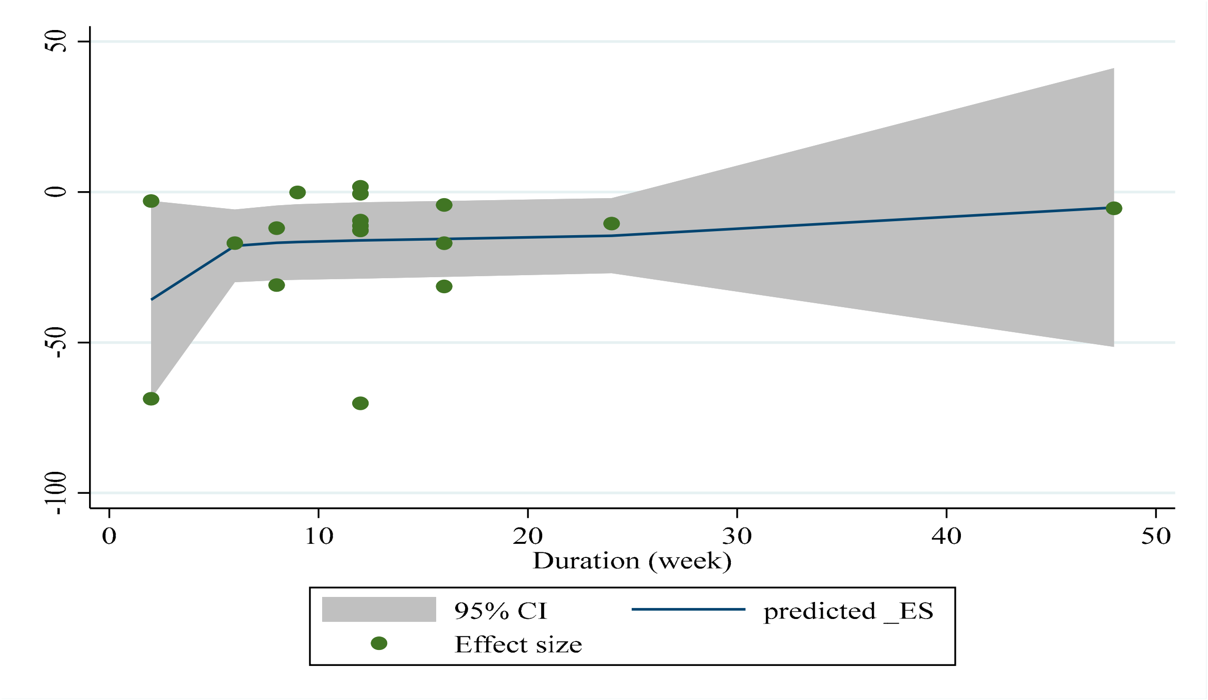

Supplementary Figure S5. *Cont.*

(G)

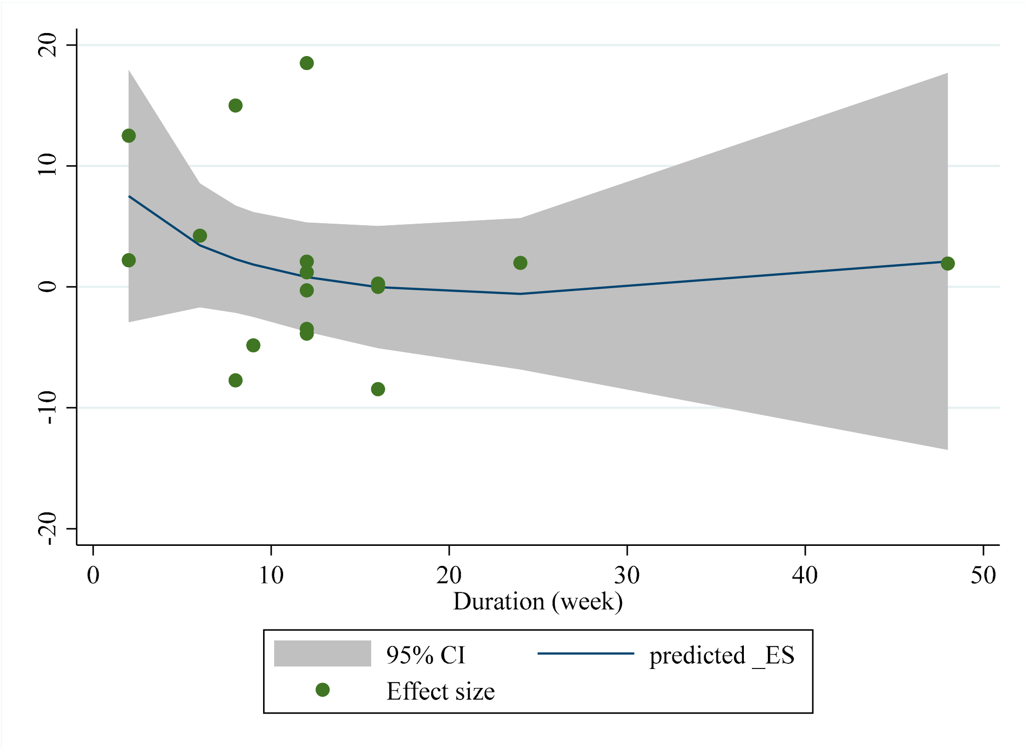

Supplementary Figure S5. Linear association between duration (week) of the supplementation with silymarin and absolute mean differences in **A)** BMI (kg/m^2), **B)** FBG (mg/dL), **C)** HbA1c (%), **D)** TG (mg/dL), **E)** TC (mg/dL), **F)** LDL (mg/dL), and **G)** HDL (mg/dL).
